# Qualitative Analyses of Textile Damage (Cuts and Tears) Applied to Fabrics Exposed to the Decomposition of Carcasses and Associated Insect Activity in an Austral Summer

**DOI:** 10.3390/insects14070618

**Published:** 2023-07-09

**Authors:** Sotirios Ziogos, Ian R. Dadour, Kari Pitts, Paola A. Magni

**Affiliations:** 1 School of Medical, Molecular & Forensic Sciences, Murdoch University, Murdoch, WA 6150, Australia; stevie.ziogos@murdoch.edu.au (S.Z.); ian.dadour@sourcecertain.com (I.R.D.); 2 Source Certain, Wangara, WA 6947, Australia; 3 Physical Evidence, Forensic Science Laboratory, ChemCentre, Bentley, WA 6983, Australia; kpitts@chemcentre.wa.gov.au; 4 Harry Butler Institute, Murdoch University, Murdoch, WA 6150, Australia

**Keywords:** taphonomy, carrion insects, degradation, homicide, stabbing, cotton, polyester, nylon, spandex

## Abstract

**Simple Summary:**

Damage on clothing worn by homicide victims can provide valuable information about the weapon or the actions of the assault. For instance, knives deposit distinct marks on fabric depending on the blade’s characteristics, and tears produce a unique morphology on clothing. However, in cases where a victim is found after a period of time, the natural processes of decomposition and insect colonization can potentially modify the damage on clothing, hindering the reconstruction of the crime. To evaluate these modifications, we conducted this study on stillborn piglets wrapped in different fabrics that were stabbed or had their fabric torn before being placed in the field to decompose. Our results show that decomposition and insect activity can modify existing cuts and tears and introduce new artifacts on fabrics as the time since death increases. The modifications of the inflicted cuts and tears were influenced by factors such as the type of fabric, type of initial damage, decomposition processes, insect activity, bacterial and fungal colonization, and length of exposure to the environment.

**Abstract:**

Fatal stabbings are the leading cause of homicide in countries with restricted access to firearms, such as Australia. The analysis of damage on clothing imparted by a sharp object can assist in the characterization of the weapon. However, decomposition and carrion insects can modify the features of the damage, impeding textile damage analysis and crime reconstruction. This study aimed to identify and characterize the modifications of textile damage over 47 days of decomposition during the summer season in Western Australia. Fabric modifications were analyzed on cotton, synthetic, and blended fabrics with standardized cuts and tears, wrapped on 99 stillborn piglets. Six unclothed piglets acted as controls, with three being stabbed. All piglets were placed simultaneously in the field alongside swatches of fabric. Analyses considered taphonomy, insect interactions, and any textile damage using optical microscopy and SEM. The results showed that carrion insects can modify existing cuts and tears and introduce new artifacts on textiles. The 100% cotton fabric was the most affected by mechanical and chemical degradation, especially cuts and areas stained with blood or decomposition fluids. The study highlights the combined effect of multiple factors on textile damage, including the type of fabric, initial damage, bloating, insect activity, and biodegradation.

## 1. Introduction

Textiles such as clothing are a common form of evidence at crime scenes and can provide valuable information in both forensic and archaeological investigations [1,2,3]. The evidential value of textiles in cases of aggravated assault is that human bodies are often clothed, and the analysis of damage found on clothing can aid in the reconstruction of events connected to a crime [4]. Injuries and fatalities caused by sharp-edged weapons often dominate statistics in countries with restricted access to firearms, such as Australia [5]. When sharp-edged weapons penetrate fabric and the skin surface, features of the action and the weapon used may be documented in the garment [1,6]. The tearing of clothing via physical force also produces characteristic features and also may have forensic value [7,8]. In natural circumstances, when a cadaver is left undisturbed on the soil surface, carrion insects colonize the remains in a predictable manner, closely linked to the particular environment and the progression of decomposition [9,10,11]. The presence of clothing or wrapping material associated with the remains may affect the pattern of insect succession, as well as the rate of decomposition, by acting as a physical barrier and restricting access [11,12,13,14]. The impact of this restriction is typically an initial delay in insect colonization, with insects eventually gaining access even to tightly wrapped remains [11,12]. At the same time, the layer of protection offered by the clothing and the increased humidity of the decomposing remains inside clothing are attractive to carrion insects such as blow flies (Diptera: Calliphoridae); ultimately, this habitat may offer a better chance of survival for immatures, especially for blow fly eggs and larvae [11,13]. As a consequence, body parts covered with clothing may allow larval masses to develop, which typically raises the temperature of the microenvironment and speeds up the decomposition process [15]. When clothing shows the presence of damage associated with a wound, the attraction of carrion insects is driven by the presence of body fluids and the easier access to the remains [13,14]. The edges of the wounds and the surrounding clothing soaked in body fluids are highly attractive to blow flies during decomposition [13]. The activity of the blow fly adults and larvae moving and feeding in and around this wound, and the associated clothing damage, may alter the relative position and the fraying of the fibers and yarns of the fabric [16,17]. When the remains reach the advanced decay and the dry stages, insect species such as larder beetles, which are attracted to hair, bones, dry skin, and clothing, become more predominant [18]; these insects generally leave clear traces of their feeding action on fabrics [19,20,21,22]. Besides acting on pre-existing textile damage, the activity of carrion insects on clothed remains has been reported to produce de novo damage or tear-like artifacts, and to be the cause of clothing disarray, which may mislead the overall reconstruction of the events that may have occurred during a criminal event [23,24,25,26]. As a result of decomposition and insect activity, the outcome of an investigation requiring the identification of a weapon based on the damage produced on the clothing of a victim can be compromised [27,28].

When human remains are in a state of advanced decomposition, there is generally a reduction in the body mass, and the damage to clothing may be the only evidence relating to the event and potential causes of death [29,30]. However, when considering damage to clothing, the correct differentiation between ante-, peri-, and post-mortem artifacts, as well as the nature of such artifacts (natural or due to a criminal action), is critical to the investigation [23,31]. Such damage identified from the clothing of a highly decomposed or skeletonized body is often the combined result of the types of fabric (natural vs. synthetic); the extent of the peri-mortem damage; and the effects of post-mortem events, such as the time of exposure, weathering, insect presence and activity, and biological or chemical degradation caused by enzymes produced by bacteria and fungi [30,32,33,34,35].

Given the large number of forensic cases involving clothed remains, analyses of fabric or textile damage are considered to be of primary importance in a criminal investigation [1,4,36]. However, at present, a limited amount of research has been conducted investigating the role of insects in the alteration of fabrics and their effect on pre-existing damage throughout the decomposition process [37]. This research presents a qualitative analysis of the impact of carrion insects on different fabrics (cut and torn) during the process of decomposition during a Western Australian summer season. The study was comprehensive, examining the extent of fabric damage resulting not only from insect activity but also the environmental conditions, microbial degradation, and fungal colonization. The main objective was to establish a baseline for the assessment of textile damage in cases involving clothed human remains and to determine whether the damage inflicted by weapons is affected by the decomposition process.

## 2. Materials and Methods

### 2.1. Piglet and Fabric Preparation

Stillborn piglets (*Sus scrofa domesticus* L., N = 105) were used as human facsimiles [38,39]. On average, the piglets weighed 2 kg, with a heart girth of 20 cm and body length of 20 cm. Pig carcasses are an appropriate alternative to humans to be used in studies focused on carrion arthropod fauna associated with decomposition [40,41]. They are also a suitable option in localities where human anthropology/decomposition facilities are not present. While larger pig cadavers are recommended, smaller cadavers may be also used [41], especially when many replicants are required.

The piglets were stored at −20 °C and thawed overnight in laboratory conditions (24 ± 2 °C) before preparation and placement in the field. The use of frozen and defrosted pigs is a common practice in forensic entomology studies, especially those that require a large number of replicants [40]. The preparation required approximately 4 h and consisted of wrapping piglets followed by stabbing or tearing the fabric. It was performed in an insect-exclusion laboratory with a constant temperature of 24 ± 2 °C. Following preparation, piglets were placed on metal trays with lids to avoid any fly colonization and then transported to the decomposition facility (approximately 1.5 km from the laboratory) using a vehicle. This was considered Day 0 (D0) of the experiment.

This study used three types of white commercial fabric, referred to in this research as “cotton” (100% cotton; no stretch), “blended” (65% polyester–35% cotton; uni-directional stretch), and “synthetic” (80% nylon–20% spandex; multi-directional stretch). Fabric details are provided in Table 1. The diameter of the fibers of the three fabrics was 23–25 μm, plied in yarns of 250 μm on average. All fabrics were initially washed together in a washing machine (3 cycles, 20 °C, powder detergent), tumble dried without heat for an hour, and then left to air dry (~25 °C). This facilitated the removal of any chemicals associated with the fabrics during production and handling. The fabrics were cut into 99 square pieces of approximately 25 cm^2^ and 36 square control swatches of approximately 10 cm^2^. All pieces of fabric were inspected for any structural damage prior to the trial.

A total of 99 piglets were wrapped in these 3 fabrics (referred to in this research as “clothed”), while 6 acted as unwrapped controls (referred to in this research as “unclothed”).

**Figure 1:**
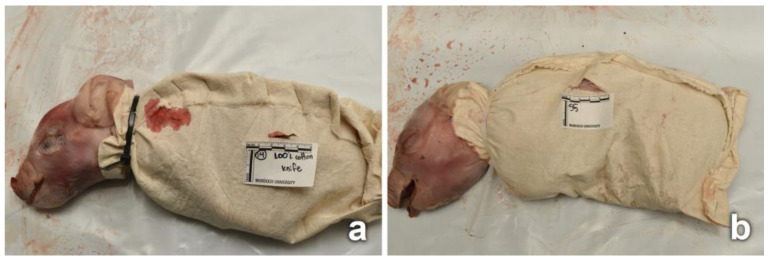
Piglets following complete preparation and before placement in the field (Cotton, D0). (**a**) Cut damage (two cuts) made by knife stab caused the fabric to be stained with blood. (**b**) Tear damage of the fabric, with piglet skin underneath intact and no blood stains.

The process of wrapping involved a piece of fabric being wrapped around the piglet, with the legs flexed under the body within the wrapping, while leaving the head exposed. The edges of the fabric were stapled together following the dorsal and caudal edges of the piglet. The fabric was secured around the neck using plastic cable ties, tight enough to secure the fabric, without inflicting damage to the neck. After the piglets were wrapped in fabric, 45 were stabbed (N = 15 wrapped in each fabric) (Figure 1a), and 45 had a tear produced in the fabric (N = 15 wrapped in each fabric) (Figure 1b). Furthermore, 9 wrapped piglets were left intact (no cut or tear, N = 3 wrapped in each fabric). Three of the 6 unclothed piglets were stabbed, and the other 3 were kept intact. All cuts and tears were produced on the left lateral side of each piglet. A summary of the experimental design is provided in Table 1.

Two stab wounds (cuts) were inflicted on each of the piglets, near the front and hind left legs, using a knife (sharp single-edged knife, with a blade length of 9 cm and a maximum width from blunt top edge (spine) to sharp edge (primary grind) of 2.2 cm). The knife was mounted perpendicularly with respect to the piglet body on a stabbing apparatus. The apparatus operated with a pneumatic solenoid valve mechanism (delivering approximately 35–40 N), producing similar stab wounds [42]. After every 30 stabbings, the knife was replaced to avoid a blunt blade or tip. The stab-produced cuts on the wrappings were approximately 2 cm long, penetrating the piglet to a depth of 4 cm. As a consequence of the stabbing, the fabric surrounding each cut became stained with blood, simulating blood seeping from wounds in real stabbing incidents (Figure 1a). A single tear in the fabric was produced between the front and hind left legs. The tear was initiated by puncturing the fabric using a blunt pair of scissors below the left front leg, followed by tearing the fabric toward the hind leg for approximately 4 cm. No physical damage was inflicted to the skin of the piglet; therefore, the fabric surrounding each tear was not stained with blood (Figure 1b).

Eight swatches each of the cotton, blended, and synthetic fabrics received cuts or tears, and 4 swatches of each fabric were not damaged. One swatch of each fabric reflecting a cut, tear, or no damage (N = 9 laboratory swatches) was kept under controlled laboratory conditions at temperature (24 ± 2 °C)/humidity (65% ± 5%), and the remaining ones were placed in the decomposition facility (N = 27 field swatches). Swatches were placed in the field in triplicate, to minimize the potential loss due to adverse weather conditions.

### 2.2. Field Setup

The decomposition facility (4H × 21W × 6D m) used in this study was in eucalypt woodland on sandy soil south of Perth city, Western Australia. The facility was surrounded by a wire fence with openings of 1 cm^2^, allowing insect entry but restricting the access of macro-scavengers such as birds, rodents, and large reptiles. The site was lightly shaded from the surrounding tree canopy and was exposed to direct sunlight for less than half the day. The facility was not used for decomposition studies for 18 months prior to this experiment, and it underwent cleaning prior to the setup.

**Figure 2:**
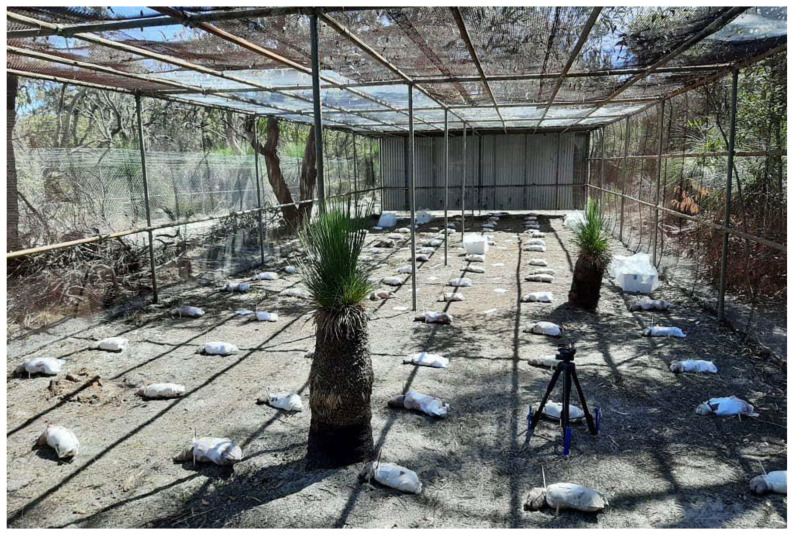
Piglet and field swatch placement in the decomposition facility used for the experiment (D0).

The 105 piglets were placed 1 m apart in a randomly generated manner (Excel^®^). Piglets were all positioned lying on their right side, with the cut or torn fabric positioned upwards. The 27 field swatches were also placed randomly, at a 50 cm distance from the piglets, pierced in one corner and tied to a pin in the ground to prevent their translocation (Figure 2). The placement of piglets and field swatches required approximately 2 h.

The trial was conducted for a total of 47 days during the southern hemisphere summer (22 February 2021 (D0)–10 April 2021 (D46)). Data loggers for temperature and humidity (Gemini Tinytag Plus-2^®^, Gemini Data Loggers Ltd, Chichester, UK) were placed in three different areas of the decomposition facility, and meteorological data comprehensive of pluviometry and wind were obtained from the closest Bureau of Meteorology station (Jandakot airport station 009172, approximately 4 km from the facility).

### 2.3. Collection and Analyses of Piglets and Fabrics

All piglets and fabric swatches were photographed daily between 9am and 12pm for the duration of the experiment. The insect activity was noted and photographed, but insects were left undisturbed until each sampling day (D6, D11, D17, D25, D46). Sampling days were chosen taking into consideration the subsequent stages of the decomposition process observed on the controls and the exposed heads of the treatment piglets, and the interaction of different groups of necrophagous insects. The final sampling day (D46) was chosen when the decomposition process was complete. The description and the allocation of the stage of decomposition was given by the external observation of each carcass. The stages of decomposition were identified as fresh, bloat, active decay, advanced decay, and skeletonized, as described by Megyesi et al. [43].

On each sampling day, 3 piglets of each combination of fabric and damage (N = 18) were chosen for collection in a randomly generated manner (Excel^®^). This was a destructive sampling design, whereby piglets, once removed, were not replaced back into the facility. This avoided any pseudoreplication of repeated sampling on the same piglets [44]. On the final sampling day (D46), controls and swatches were also recovered.

Piglets were photographed in situ before and after the removal of the fabric; insects were sampled from the piglets, the fabric, and the surrounding soil, using the guidelines of best practice in forensic entomology [45]. Following collection, 50% of the sample was preserved [45], and 50% was reared in the laboratory for morphological identification purposes [11]. Blow flies (adults and larvae) were identified using the key of Wallman [46], the beetles using the key of Lawrence et al. [47], and the remaining insects using CSIRO Insects of Australia [48].

Fabrics from each piglet were photographed in situ using a scale. This included close-ups of the cuts or tears as well as any noted artifacts. The fabric was then removed and placed flat, covered with air-permeable paper, placed in a paper envelope, and allowed to air dry in the laboratory prior to examination. Similarly, field swatches were also photographed in situ daily, and, on the final sampling day, they were collected and preserved in paper envelopes.

All pre-existing and new fabric damage was assessed initially by examining the yarn and fiber structure [36] using a digital microscope (Dino-Lite^®^ edge 3.0 paired with Dino-Capture^®^ software, Dunwell Tech., Inc., Torrence, CA, USA), stereomicroscope (Leica^®^ M205C, Leica Microsystems, Wetzlar, Germany), and compound microscope (Leitz-Diaplan^®^, Leitz, Wetzlar, Germany). Inflicted cuts and tears were compared to the corresponding control field and laboratory swatches. All new artifacts were examined, with fiber samples taken from areas of suspected insect damage and insect-induced modifications in the vicinity of the cuts and tears. The morphology of the fiber ends of each cut and tear was examined using scanning electron microscopy (TESCAN MIRA4 FE-SEM^®^, TESCAN, Brno, Czech Republic).

## 3. Results

Due to the experimental design involving the decomposition of many carcasses and several treatment groups, the results of this research are categorized into sections. These sections include analyses of environmental conditions, the process of decomposition, insect assemblage, and data, with the Supplementary Materials providing an overview of the fabric damage modifications. The Discussion section will explore the respective impacts and interactions among these variables, providing a comprehensive examination of our findings.

### 3.1. Environmental Conditions

Overall, the weather conditions throughout the trial were hot and dry, and most days were sunny, with a day length of approximately 13 h. The average daily temperature, based on data collected from three separate data loggers, was 23.0 ± 5 °C, with the maximum recorded temperature on D35 (38.6 °C) and minimum temperature on D30 (10.0 °C). The average relative humidity was 61.0 ± 20.3%, with a maximum of 95.3% on D18 and a minimum of 11.5% on D25. The accumulated precipitation was 35.5 mm over the 47 days of the trial, with maximum daily rainfall of 9 mm on D6 and 17.7 mm on D7. The average wind velocity was 19.5 km/h, with average maximum wind gusts of 41.6 km/h and the highest recorded wind gust of 65.0 km/h on D8.

### 3.2. Decomposition Process

**Figure 3:**
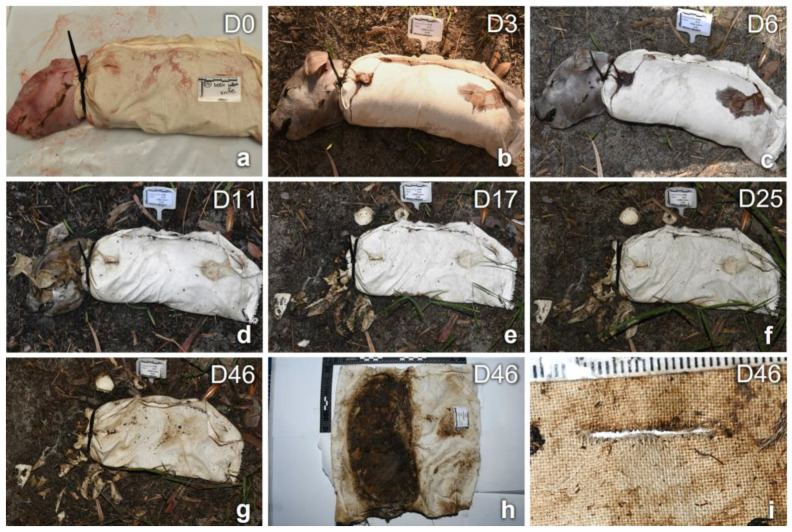
Process of decomposition of piglet replicant #15 (cotton, D0–D46, cut) on the days of placement, maximum bloating, sampling, and final recovery (**a**–**i**). Particulars of the whole fabric and the damage are provided in (**h**,**i**). Throughout the decomposition process, it was possible to appreciate the fresh stage (**a**), maximum bloating and misalignment of the wound/fabric damage (**b**), the formation of a small larval mass of the head (**d**), differential decomposition (**e**), bone scattering (**e**–**g**), and larvae at the edges of the cut closer to the head. The position of the remains could be inferred from the recovered fabrics (**h**). The recovered cuts appeared affected by bloating and the overall process of decomposition and appeared distorted on the day of collection, D46 (**i**).

Unclothed piglets underwent a process of decomposition involving the whole body, which was observed on a daily basis, whereas clothed piglets were unwrapped only on collection days; therefore, the stages of observed decomposition pertained only to the visible part of the body (head). An overview of the decomposition process from D0 to D46 of a stabbed piglet clothed in cotton fabric is illustrated in Figure 3.

Overall, the unclothed piglets decomposed faster compared to the clothed piglets, with the skeletonization of the whole body starting as early as D16 for unclothed piglets as compared to D19 for the heads of piglets clothed in blended fabrics, D27 in synthetics, and D33 in cotton.

In general, piglets entered the bloat stage by D2, with most of them passing to active decay between D6 and D9. Advanced decay was observed from D17. While the skeletonization of unclothed piglets was of the overall body at the same time, in clothed piglets, the head area skeletonized first due to the large larval masses present during the advanced decay stage. Complete skeletonization was reached by all piglets on D46.

When considering all the clothed piglets observed daily over the entire trial, no differences in the speed of decomposition were noticed between stabbed and intact piglets; however, piglets clothed in synthetic fabric showed prolonged wet stages (active + advanced decay) as compared to piglets in cotton and blended fabrics.

### 3.3. Entomological Data

The insects and other arthropods collected from the control piglets and the clothed piglets following the removal of the fabric on collection days are reported in Table 2. Based on the observations and insect samples collected, differences were observed between clothed and unclothed piglets, but not within different treatments of clothed piglets; therefore, the data regarding the clothed piglets were combined.

**Figure 4:**
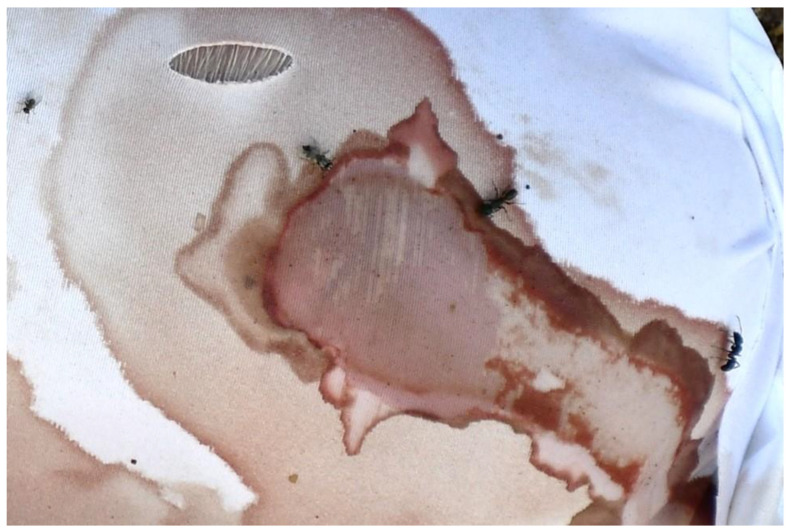
Stabbed piglet in bloat stage (synthetic, D2). Notice adult insects visiting the bloodstained areas and the misalignment of cuts on the wrappings with respect to the underlying wound as the result of bloating.

Adult blow flies were the first carrion insects attracted to all piglets, arriving on D0 following the placement of the carcasses, and were collected from D6. Other fly and beetle species, as well as other groups, were observed during advanced decay and collected from D11. Colonization by blow fly immatures was observed on all clothed piglets from D1, but mainly around the natural orifices of the head and, for the unclothed piglets, also on the umbilical, the genital, and the stab wound regions. The body parts exposed by cuts and tears (lesions or intact skin) were an attractive area for insects to frequent, visiting and moving in and around the fabric damage. Adult insects from the orders of Diptera, Coleoptera, and Hymenoptera (ants) were observed, particularly in association with the stab wounds and bloodstained areas of fabric (Figure 4). Insects were feeding and gaining access to the carcass through the damaged fabric until the first day of rain (D6).

**Figure 5:**
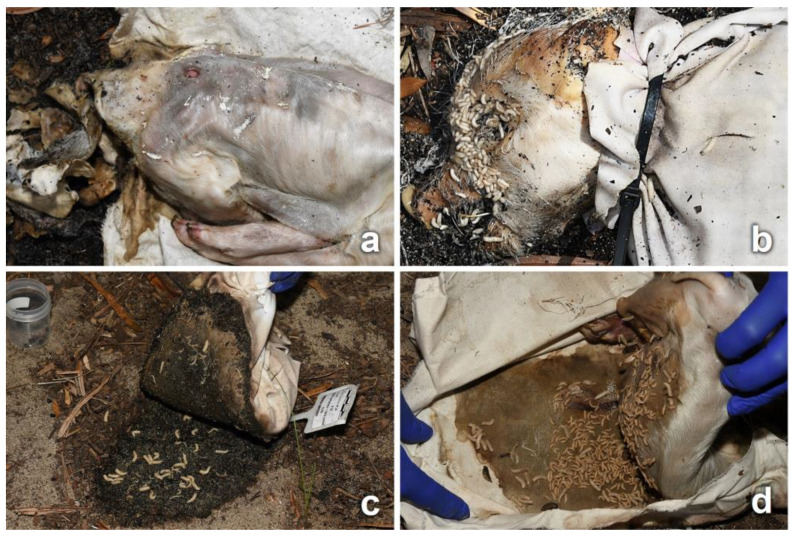
(**a**) Egg cluster depositions observed on the skin of a piglet on collection day (cotton, D11, cut). Differential decomposition between the head and the body can also be observed, with the head being completely skeletonized. (**b**) Larval mass on the head of a piglet (synthetic, D11, cut). Blow fly larvae can also be observed moving through the cut. (**c**) Blow fly larvae observed in the interface carcass/soil on collection day (blended, D11, cut). (**d**) Larval mass observed inside a cotton wrapping damaged with a tear (D11).

On all piglets, egg clusters were observed primarily around the natural orifices of the head, and on the genitalia where exposed. No egg clusters were observed on the fabric cuts and tears; however, when the fabric was removed on D6, empty egg clusters were observed distributed across the skin covered by the fabric (Figure 5a).

**Figure 6:**
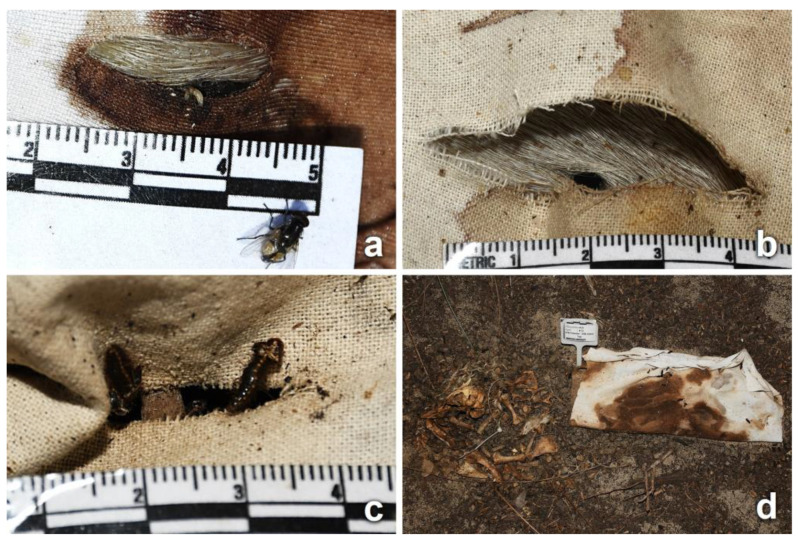
(**a**) Fly larvae interacting with yarns and fibers at the immediate area of a bloodstained cut (synthetic, D5). (**b**) Entrance/exit hole on exposed skin observed during active decay on D11 through a tear on cotton fabric. (**c**) Dermestid immature and exuviae observed on a cut (cotton, D46). (**d**) Displacement of bones of skeletonized remains. Dermestid adults and immatures, pupal cases, and ants observed on the fabric (blended, D46, tear) and the surrounding area. The soil immediately surrounding the remains had a powdery appearance and ant nests could be observed nearby.

As the decomposition process advanced, large larval masses were observed in the eyes, inside the mouth (Figure 5b), around the genital area, and at the interface of the carcass with the soil (Figure 5c). On clothed piglets, the movement of the fabric was indicative of the presence of active larval masses at the interface between the carcass and fabric, although direct observation was only possible on the day of collection (Figure 5d). Fly larvae were observed moving through the cuts and tears, primarily on stained areas (Figure 5b and Figure 6a–c). Symmetrical round holes that may have been caused by fly larvae, and/or by dermestid (Coleoptera: Dermestidae) larvae and adults during the later stages of decomposition [11], were observed on the skin of clothed and unclothed piglets from D10 onwards. On clothed piglets, these holes were observed on the skin surrounding the stab wounds or on skin exposed through tears (Figure 6b). In unclothed piglets, the holes were observed in several locations of the upper body.

Towards the later stages of decomposition, immature and adult dermestids, remnants of past insect activity including powdery dermestid frass and exuviae, and full and empty pupal cases were observed on the different types of fabrics and in the soil surrounding each carcass (Figure 6d). Ant nests were noticed near several of the piglets, with both ants and rove beetles predating on fly larvae (Figure S8 in Supplementary Materials).

In total, twenty insect taxa were collected and identified from the decomposing piglets and the surrounding soil during the trial (Table 2). Based on the samples collected and the photographic material produced, the taxa colonizing clothed and unclothed piglets were similar in terms of the species and pattern of colonization, irrespective of there being cuts or tears.

Nine species of Diptera colonized the piglets, including four species of blow flies. *Chrysomya rufifacies* Maquart (Diptera: Calliphoridae) and *Ch. varipes* Robineau-Desvoidy, which were the initial and predominant colonizers throughout the trial, visited from D0 and oviposited from D1. *Lucilia sericata* Meigen (Diptera: Calliphoridae) and *Calliphora dubia* Maquart (Diptera: Calliphoridae) adults were commonly observed at the beginning of the trial, and larval samples were collected up to D11. Colonization by *C. dubia* was less extensive than that of *Ch. rufifacies*, *Ch. varipes*, and *L. sericata*. Blow fly pupal cases were observed starting from the D11 collection, some of which were parasitized by the parasitoid wasp *Nasonia vitripennis* (Walker) (Hymenoptera: Pteromalidae) [49,50,51].

Flesh flies (Diptera: Sarcophagidae) were also observed to colonize and larviposit from D2. Third-instar sarcophagid larvae were collected on D6 from most piglets, but also on D25, especially from piglets clothed in synthetic fabric. Adult muscid species, *Musca domestica* L. (Diptera: Muscidae) and *M. vetustissima* Walker, were observed to be low in abundance throughout the trial; a small number of third-instar larvae were collected on D6 from piglets clothed in cotton and blended fabrics, and from all piglets from D11 onwards. Adults of phorid flies (*Megaselia scalaris* (Loew), Diptera: Phoridae) were observed on four piglets from D11, and a small number of full pupal cases were collected from piglets clothed in cotton and blended fabrics on D17 and D25. Adults of *Fannia canicularis* Robineau-Desvoidy (Diptera: Fanniidae) were first observed on D11, with larval and pupal specimens collected from all fabric types of clothed piglets (N = 9). The population of *F. canicularis* increased from D17 until the end of the trial.

Five taxa of Coleoptera were collected throughout the trial. The first observed were clown beetles (*Saprinus* spp., Coleoptera: Histeridae) on D5, with adults collected from all clothed piglets from D6 and throughout the experiment. However, this species was not observed or collected on unclothed piglets after D11. The population of dermestids (*Dermestes* spp., Coleoptera: Dermestidae) increased and became the predominant colonizer on all clothed piglets during the stage of advanced decay and the early stages of dry decay (D11 to D46). In comparison, only a small number of dermestids were collected from unclothed piglets. Rove beetles (*Creophilus erythrocephalus* Fabricius, Coleoptera: Staphylinidae) and checkered beetles (*Necrobia rufipes* (De Geer), Coleoptera: Cleridae) were observed and collected in low numbers on D11 onwards and only from unclothed piglets.

In addition, ants (Hymenoptera: Formicidae) played a role in the decomposition process, infesting a large number of piglets from D1 and throughout the trial [52].

Furthermore, several taxa of incidental arthropods were also observed and collected. These included *Omorgus tatei* Blackburn (Coleoptera: Trogidae), commonly found in Australia, feeding on the dung of various animals [48], and were collected in small numbers, alongside mites (Class Arachnida), spiders (Order Araneae), earwigs (Order Dermaptera), millipedes (Class Diplopoda), and centipedes (Class Chilopoda).

### 3.4. Observations on Fabrics

The morphology of the cuts and tears on the field and laboratory fabric swatches was similar, with the slight fraying of yarn ends in the field swatches. Overall, no new damage was observed on any of the field or lab swatches by D46 (Figure S1 and S2 in Supplementary Materials).

**Figure 7:**
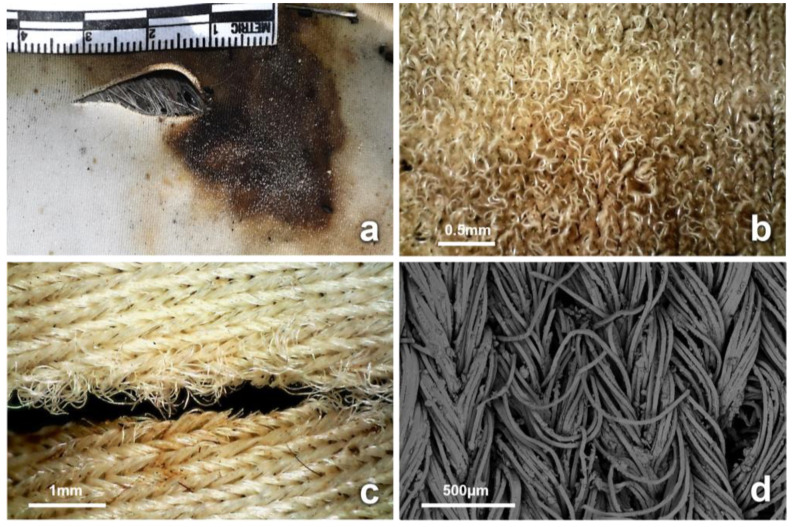
(**a**) Increased fraying of the surface was observed on the bloodstained area (synthetic, D11, cut). (**b**) Surface damage on synthetic wrappings showed fibers that appeared pulled out of the fabric structure. (**c**) Insect activity on the cuts also resulted in damage to the fabric surface, with displacement of fibers and increased fraying. (**d**) SEM micrograph of fiber specimens taken from areas of surface damage not associated with inflicted damage that appear displaced (synthetic, D46).

In the fabric wrappings, variations in the morphology and dimensions of all initial cuts and tears were observed during the process of decomposition. The bloating of the carcasses resulted in the misalignment of the fabric cuts with the underlying wounds (Figure 4) and the stretching of the damaged fabric area (Figures S3 and S4 in Supplementary Materials). Sections of most cuts were stained with blood from the bleeding stab wounds. Insects were observed interacting with the bloodstained areas of fabric (Figure 4). Artifacts and modifications at the structural level of previously intact areas of fabric (not linked to the initial cuts or tears) were observed on more than half of the recovered wrappings, regardless of treatment. These new artifacts were observed as early as D6, and they were classified based on their morphology in terms of surface distortion and holes. Distortion on the fabric surface was observed in areas that had been stained with blood. The bloodstained areas showed distortion of the fabric structure (knit or weave), with distorted yarns, pulled fibers, and fractures along the fiber axes and at the fiber ends (Figure 7 and Figure S9 in Supplementary Materials). In total, 40 distinct areas of surface distortion were identified on 8 cotton, 13 blended, and 16 synthetic fabrics.

**Figure 8:**
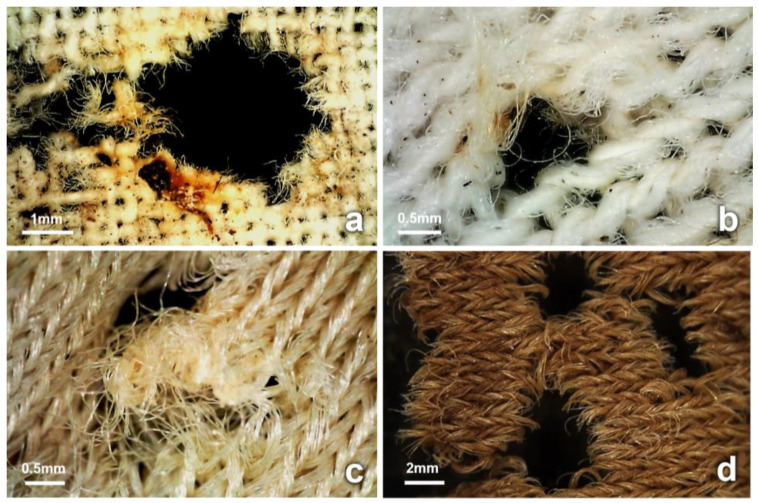
New artifacts on previously intact and stained areas of fabric observed on wrappings recovered on D46. (**a**) Artifact with brittle fractures on cotton fabric. Insect excreta and dermestid activity were observed at the area of the depicted artifact. (**b**) Stretched yarn intersection and pulled out yarns of blended fabric could possibly be the result of insects passing through the knit and could potentially appear as a puncture artifact. (**c**) Hole on synthetic fabric with localized fraying on the fabric surface that could resemble puncture-like or tear-like characteristics. (**d**) Fabric attrition on synthetic wrapping with signs of deterioration accompanied by localized surface fraying.

Holes of sizes ranging between 2 and 81 mm (maximum end-to-end distance), with various degrees of fraying yarns and limited signs of the chemical degradation of fibers, were observed primarily on stained fabric areas from D6 and throughout the trial (Figure 8). These holes appeared to have been formed by the rupturing of adjacent yarns, often associated with the distortion of the fabric surface. In total, 39 holes were located on 12 cotton, 6 blended, and 6 synthetic fabrics. Stretched intersections of yarns with increased fraying and pulled fibers due to the tightening of adjacent yarns were also observed, primarily on cotton fabrics.

**Figure 9:**
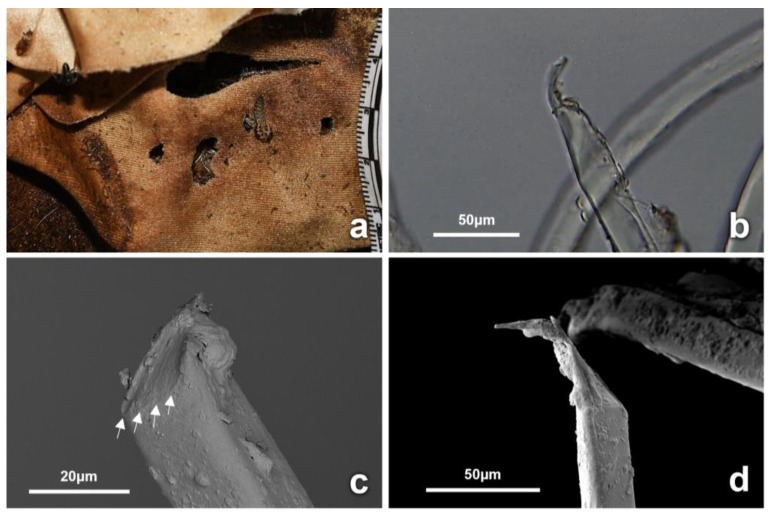
(**a**) Dermestid larvae, exuviae, and new artifacts can be observed on a stained wrapping proximal to the inflicted cut (synthetic, D46). The cut appears modified, with missing sections, and accompanied by new artifacts possibly inflicted by the observed activity of dermestids at this area. (**b**) Microscopic examination of nylon fiber specimens taken from the artifacts stained with decomposition material revealed concave-shaped fractures at the fiber ends. (**c**) SEM micrograph of stained nylon fiber specimen taken from a new artifact. The structure of the fiber end appears mechanically altered and curved. The fracture appears defined with striations (highlighted with arrows), possibly deposited from an insect appendage. (**d**) SEM micrograph of uncut nylon fiber, with fiber end morphology that reveals a defined flat end, potentially resembling fiber severance resulting from a blade.

In addition, holes with fractured yarns, characteristic fiber fractures, and cracks along the axes were observed. The fiber ends surrounding the holes appeared often to be concave or curve-shaped, with and without striations (Figure 9c,d). These artifacts were observed on dry fabric areas stained with decomposition fluid from D25 only; they were first noticed on a single cotton fabric, but then observed on 14 fabrics of all types during the dry stage of decomposition (Figure 9 and Figure S10 in Supplementary Materials).

Fabrics collected on D46 showed many holes, especially in areas of interface with the soil, and incorporated decomposition fluid and soil (Figures S6 and S7 in Supplementary Materials). The degraded areas followed the outline of the carcass and were observed primarily in cotton fabrics. Microscopic examination and SEM analysis of the fibers revealed the chemical degradation of the affected yarns and fibers, particularly at the ends. This occurred on a total of 20 fabric samples, 14 of which were cotton, 4 synthetic, and 2 blended.

Holes with discoloration and a notably darker appearance of the yarn ends were observed at several areas of the fabric and not necessarily in contact with the soil. These holes were of several different sizes and observed on stained and dry areas of fabric. SEM examination showed mold growth, with yarns and fibers having the appearance of chemical breakdown. The first hole with mold colonization was observed on D6 on a cotton wrapping. Mold colonized a total of 13 areas, 10 of which were observed on cotton and three on synthetic fabrics (Figure S7 in Supplementary Materials).

## 4. Discussion

The present research was conducted using three types of fabric to wrap stillborn piglets, which were then left decomposing in the Australian bushland during the summer season. These piglets reached skeletonization by day 46, in contrast to the 98+ days for 2-year-old pigs (~40 kg) used in similar experiments in the same region and time of the year [53].

### 4.1. Inflicted Damage (Cuts and Tears)

When comparing the observed modification of the cuts and tears throughout the 47-day trial, cuts that were associated with stabbing and then stained with biological material were affected most noticeably, especially the cotton fabric. Overall, the modification of the cuts and tears was the result of the combined effect of different factors and events related to the decomposition process (Figure S5 in Supplementary Materials). These factors included the mechanical stretching of the fabric during the bloat stage, staining with blood and decomposition fluid, insects visiting and feeding on the blood stains produced by the underlying wounds, mechanical and chemical degradation due to microbial and fungal growth, and the time of exposure. As a result, added fraying, distortion of the edges, and modifications in the planar array of cuts were introduced. These factors are indicative of tearing [54,55] and affect the estimation of blade sharpness, and they generally may limit the information that can be extracted [54]. Sharp blades will typically cut a fabric with the local application of pressure to the fibers, resulting in smooth and flat yarn ends seen as a planar array. Duller blades may cause stretching and tearing, resulting in distortion and frayed yarn ends, similar to serrated blades [6]. In this study, after 46 days of exposure, most cuts could still be indicated, although the extraction of other blade characteristics was problematic.

On the contrary, yarns and fibers of unstained sections of cuts on D46 had retained some characteristics, such as the alignment of flat yarn ends (planar array) and the presence of yarn loops (snippets) within cuts [36]. Concurrent with this, no signs of modification were observed on field swatches of any fabric type by the end of the trial (Figures S1 and S2 in Supplementary Materials).

### 4.2. Insect-Induced Modifications

Overall, the interaction of carrion insects between the carcass and the fabrics can be ascribed to two types: the close presence of necrophagous activity of feeding on the remains and the decomposition fluids that seeped into the fabrics and the associated activity of insects feeding directly on the fibers. The interaction of insects with the fabrics resulted in the mechanical modification of the fabric structure, including yarns and fibers.

The first activity type involved the effect of necrophagous insects on the yarns, causing a weathered and abraded appearance, with significant fraying and displacement of yarns and fibers at the fabric surface, especially around cuts stained with blood (Figure 7 and Figure S9 in Supplementary Materials). This may be a result of adult blow flies feeding on liquids during all stages of decomposition, using sponging and sucking mouthparts, as well as enzymes that may be egested when feeding [56]. In addition, the sponge-like labella have structures such as prestomal teeth and labellar hooks, able to abrade the skin and open wounds in order to feed [57]. In carrion blow flies such as *C. vomitoria* and *L. sericata*, the prestomal teeth have a width of approximately 15 μm and are highly sclerotized, with various degrees of serration. In addition to this, the scraping action of the labellum has been reported to be able to cut through 50 μm of tissue [58,59,60]. The combined action of the prestomal teeth and labellar hooks on bloodstained fabrics may result in the fracture and displacement of yarns and the overall distorted appearance of the fabric structure. Cotton fabrics were more fractured and brittle, with more missing fibers, than blended and synthetic fabrics [61]. Generally, yarn fraying and the displacement of fibers on a fabric surface are caused during wear, washing, and rubbing [61,62]. The results of this research show that alterations caused by insects on the surfaces of fabrics during the decomposition process may mimic abrasive types of damage.

Fabric distortion may also be caused by the movement of blow fly larvae and their sclerotized mouth hooks, which assist in crawling and rasping the tissues of the piglet, or the fabric soaked in body fluids [11]. This was evident in this trial, with larvae observed passing through the fabric cuts and tears and feeding at the wound sites (Figure 5b and Figure 6). Newly hatched larvae have been reported to crawl through the weaves of fabrics [11], and this observation is consistent with the stretched knit and weave intersections that were observed on fabrics (Figure 8b). The mouth hooks of third-instar *Ch. rufifacies* are approximately 100 μm, and each mouth hook is serrated, with several rows of pointed margins [63,64]. Many insect larvae also possess adhesive hooks and spear-like setae that grip and detach from the substrate to assist in forward motion [57]. Large larval masses of more than 1000 third-instar individuals can potentially be even more damaging to textiles through cooperative foraging and the frenetic movement of larvae, especially when their bodies have protuberances and spikes, as in *Ch. rufifacies* [65,66].

Further alterations of the fabric surface and ruptured yarns are possibly caused by insects feeding and moving through the stained yarns. This was first observed on cotton from D6, especially around stained fabric areas (Figure 7 and Figure 8), and appeared to be affected by the weave density, elasticity, and fabric structure. In this study, cotton had the lowest density when compared to blended and synthetic fabrics. The morphology resulting from this activity resembled small holes and weathered puncture marks, similar to those made by piercing instruments such as screwdrivers, needles, and syringes [36,54]. When moving across fabrics, adult insect legs possess claws, pulvilli, and tenent hairs to assist in movement and other functions [57]. Furthermore, adult blow flies are equipped with a pair of large tarsal claws and pulvilli with spatula-like tenent setae that assist when attaching to surfaces. These tarsal claws can be almost 250 μm in length, with many spines and a ribbed distal tip, which are important when clasping and adhering to irregularities in surfaces [67,68], and these may alter the structures of fabrics.

The second type of activity of insects is those feeding directly on the fabrics, which can cause fiber fractures. In this trial, fractures often concave in shape were observed along the yarns and at the fiber ends. The morphology of the ends of many fibers was flat as well as striated, which may have resulted from insect appendages. These flat fiber ends may simulate the morphology of severed fibers caused by a blade. However, this was observed on individual fibers only in a disorganized manner and did not simulate the fiber evenness typically seen on yarns cut by a blade. Such characteristics often appeared as parts of holes on dry areas stained with decomposition fluid, first observed on cotton on D25 and subsequently during the drier stages of decomposition. Dermestid larvae and adults were often observed interacting with areas of fabric and the cuts stained with decomposition fluid (Figure 6 and Figure 9). Insects that feed in later stages of decomposition, such as dermestids, have mandibulate or chewing mouthparts designed to cut and crush their food items in a transverse action [57]. These characteristic features were evident on the fibers of the different fabrics (Figure 9). Anderson et al. characterized dermestid bites on wool fibers by *Anthrenus vorax* (LeConte) (Coleoptera: Dermestidae) as sharply defined bites [69]. Dermestid larvae, earwigs, and ants also have chewing mouthparts, all of which were collected during this trial and often seen associated with the stained fabrics. The concave-shaped fractures of fibers in this trial were consistent with fractures caused by dermestid larvae on proteinaceous fibers and human hair in other studies [69,70,71,72]. This is also supported by Mallis et al. [20], who reported that dermestid larvae were observed feeding on nylon and polyester fibers stained with human sweat and urine. Similarly, Finley et al. [21] investigated damage caused to fabrics stained with animal fat by pest insect species and showed that the damage was consistent with dermestid activity on stained synthetic fabrics.

### 4.3. Biodegradation

New artifacts on wrappings were observed along the periphery of the decomposed carcass from D46, mainly on cotton, where the decomposition fluids had leached through the fabric and had become dry but remained in contact with the soil (Figure S6 in Supplementary Materials) [34]. The morphology of the fibers examined with SEM appeared consistent with biodegradation, with a “melted” or “softened” appearance. The biodegradation of textiles is damage caused by the biological attacks of enzymes secreted by bacteria and fungi and is normally gradual [31].

The present study demonstrated that fabric degradation was less evident at the interface between the carcass and soil. The fabric in these areas was often embedded with dried decomposition material and soil debris. This observation is in agreement with the findings of Ueland et al. [35] and Lowe et al. [73], who reported that clothing may be preserved when partially or completely buried in soil and in contact with a decomposing pig, where decomposition fluids stagnate. Furthermore, holes were observed in the fabric at the interface between the carcass and the soil (Figure 5c and Figure S6b in Supplementary Materials) [11]. Fly larvae and beetles were frequently observed near these areas, but, due to the presence of debris material embedded in the edges of the holes, there was insufficient evidence that these holes were originally caused by insects or that insects used these holes to access the remains.

The blood and decomposition fluid represented favorable substrates for fungal growth, which consequently promoted the mechanical and chemical degradation of fibers [74,75]. Fungal spores can be conveyed by the wind, contact with the soil, and the activity of visiting insects. Flies have been shown to be responsible for the mechanical transmission of soil microbes, viruses, bacteria, and fungi from one site to another following egestion, and the direct contact of the labellum, prestomal teeth, and legs with a substrate [56,67]. The evidence of cracks and fractures on fibers via fly appendages, and the transmission of microbes to these sites, could serve as points for further microbe growth on fabrics [71,75,76]. These may cause mechanical and chemical alterations to the fibers, especially on stained areas of recovered clothing during the decomposition process. However, due to the complexity of the overlapping damage on different types of fibers [77,78], SEM was unable to determine whether this was caused by insect activity (Figure S10 in Supplementary Materials).

## 5. Conclusions

The forensic significance of textile damage is reflective of its ability to capture the features of the implement(s) or actions involved. When undertaking a textile damage assessment of a garment from a decedent, the contribution attributed to decomposition and associated factors may obscure the exact nature of how the fabric was damaged. In this study, damage on recovered fabrics varied. Factors that promoted the degradation of fabrics were the fabric type, blood, other decomposition fluids, the location of the damage in relation to the position of the carcass, insect activity, biodegradation, and the time of exposure. Therefore, these factors can obliterate fabric characteristics that may be of forensic significance.

The importance of the systematic recording of textile damage, both qualitatively and quantitatively, to ensure accurate interpretation has been emphasized in a few studies [8,54,79] and outlined by the Textile Damage Working Group of Australia and New Zealand [8,80]. This study provides a detailed description of qualitative assessments associated with decomposition that can modify the fabric structure and alter the appearance of textile damage in these fabrics. The misinterpretation of observational data in cases where multiple types of damage are present can hinder the examination, limiting the reliability of the evidence and the eventual interpretation.

Therefore, it is recommended that investigators use caution when classifying cuts or tears in fabric recovered from a highly decomposed or skeletonized body. It is noted that unstained areas are more likely to retain indicators of the cause of textile damage. Focusing on unstained areas can avoid the interpretation of alterations caused by carrion insects attracted to stained areas but is unlikely to be practical in casework. It is clear that more research is required to bolster the body of knowledge in regard to the interaction of decomposition with textiles of any type in a forensic context.

Furthermore, in a quantitative study, another control should be considered, namely piglets wrapped in fabrics and insects excluded. To accomplish this, many other environmental variables would need to be excluded. However, in this study, it was unnecessary as we focused on insect damage to the fabrics. With insects excluded from the decomposition process, there would be no insect damage to fabrics, similar to those swatches of fabric that had no association with piglets placed in the facility.

One other matter that should be addressed is the size and the distance between the piglets. According to Michaud et al. (2012) and Boudreau and Moreau (2022), 50 m between carcasses has “more or less emerged” as an optimum distance to arrest cross-contamination by larvae, as well as several kilometers for adult flies [81,82]. However, other studies have investigated the effect of the distance between cadavers in forensic entomology and taphonomy [83,84,85,86,87]. One of these studies, which is a review by Tomberlin et al. (2017) [87], detailed that when there is a high concentration of carcasses in a small area, invertebrate consumers, such as blow flies, can rapidly increase in abundance due to their reproductive capacity. Furthermore, these authors suggested that the efficiency of invertebrates to colonize and decompose vertebrate carrion decreases as the amount of carrion in a location increases [87]. As a consequence, it is possible that the pattern and rate of insect-mediated decomposition could be influenced by cross-contamination effects when there are small distances between small cadavers. However, Boudreau and Moreau (2022) stated that in the experimental context of forensic entomology, the potential impact of small-scale factors can only be assumed to introduce potential bias to the information, due to a lack of definitive research [82]. In the context of this study, such concerns were not significant, as the primary focus was on the qualitative examination of whether insects can modify existing damage to fabrics associated with decomposing cadavers.

## Figures and Tables

**Table 1:** A summary of the experimental design. Fabric type per damage type per number of piglets and fabric swatches; n/a: not applicable (in treatments with naked pigs no fabric swatches were associated with naked piglets).

Fabric Type and Characteristics	Damage Type	Number of Piglets	Number of Swatches
100% cotton; Natural; Plain weave (1:1); No stretch	Cut	15	4
	Tear	15	4
	Nil	3	4
65% polyester–35% cotton; Blend (synthetic/natural); Jersey knit; Uni-directional stretch	Cut	15	4
	Tear	15	4
	Nil	3	4
80% nylon–20% spandex; Synthetic (all-synthetic blend); Jersey knit; Multi-directional stretch	Cut	15	4
	Tear	15	4
	Nil	3	4
Unclothed; (No fabric)	Cut	3	n/a
	Nil	3	n/a
Total	-	105	36

**Table 2:** Insects and other arthropods collected from both unclothed and clothed piglets following the removal of the fabric on collection days. UCC = unclothed control, AFT = all fabric types, E = eggs, L = larvae in unspecified stage, L2 = larvae in second stage of life, L3 = larvae in third stage of life, PF = post-feeding larvae, P = pupae (full pupal case), Pu = puparia (empty pupal case), PP = parasitized pupal case, A = adult; n/a = not applicable (from D25 no insects were collected from unclothed controls).

Order	Family	Species	Day 6		Day 11		Day 17		Day 25		Day 46	
			UCC	AFT	UCC	AFT	UCC	AFT	UCC	AFT	UCC	AFT
Diptera	Calliphoridae	*Chrysomya* *rufifacies*		L1, L2, L3	L3	L3, P		L3, P, PF	n/a	P, Pu	n/a	P, Pu, PP
		*Chrysomya varipes*		L1, L2, L3		L3, P		A, L3, P		P, Pu		P, Pu, PP
		*Calliphora dubia*	L2, L3	L2, L3		L3	P			L, PP		P
		*Lucilia sericata*		L2, L3		L3						
	Sarcophagidae	spp.	L3	L3						L3		
	Muscidae	*Musca* *vetustissima*		L3	L3	L3	L3	L3, PF, P		L3, PF, P		L3, PF, P
		*Australophyra rostrata*		L3	L3	L3, A	L3	L3, PF, P		L3, PF, P		L3, PF, P
		*Musca domestica*		L3	L3	L3, A	L3	L3, PF, P		L3, PF, P		L3, PF, P
	Phoridae	spp.				A	P	P		P		
	Fanniidae	*Fannia* *canicularis*				A, L, P	L	A, L, P		A, L3, P		L
Coleoptera	Dermestidae	*Dermestes* spp.				A	L	A, L, P		A, L		A, L, P
	Histeridae	*Saprinus* spp.	A	A	A	A		A		A		A
	Staphylinidae	*Creophilus* *erythrocephalus*				A		A		A		A
	Cleridae	*Necrobia rufipes*				A		A		A		A
	Trogidae	*Omorgus tatei*								A		
Others			E	E, ants, earwigs		Millipedes, mites, ants		Mites, ants		Mites, ants, dung beetles		Millipedes, mites, ants, earwigs

## Data Availability

All data are contained within the article and the Supplementary Materials.

## Supplementary Materials

The following supporting information can be downloaded at: https://www.mdpi.com/article/10.3390/insects14070618/s1. Figure S1, Side by side comparison of cuts and tears in lab and field swatches on D46; Figure S2, Photographs comparing cuts and tears in lab and field swatches and textile damage on wrappings recovered on D46 for the three fabric types; Figure S3, Photographs of a wrapped piglet before and during the bloat stage; Figure S4, Photographs highlighting the modifications to the inflicted fabric damage (cut or tear) on wrappings after the bloat stage and during the process of decomposition; Figure S5, Modifications observed on stained cuts recovered on D6, D11, D17, D25, and D46; Figure S6, Degradation of cotton wrappings; Figure S7, Photographs, micrographs and electron micrographs of artifact-damaged yarns and fibers; Figure S8, Ants and opportunistic insects observed from D2 and throughout the trial; Figure S9, Distortion of stained fabric surface; Figure S10, New artifacts (holes) on a stained and previously intact area.
