# Supplementary material for: Qualitative Analyses of Textile Damage (Cuts and Tears) Applied to Fabrics Exposed to the Decomposition of Carcasses and Associated Insect Activity in an Austral Summer
Source: Insects. 2023 Jul 9;14(7):618. doi: 10.3390/insects14070618 (PMC10380818; doi:10.3390/insects14070618)
Supplement: Supplementary file 1 [file insects-14-00618-s001.zip › insects-2403833-supplementary.docx]

Supplementary Materials of the Article:

Qualitative analyses of textile damage (cuts and tears) applied to fabrics exposed to the decomposition of carcasses and associated insect activity in an Austral summer

Sotirios Ziogos ^1^, Ian R. Dadour ^1, 2^, Kari Pitts ^3^ and Paola A. Magni ^1, 4^ *

^1^ School of Medical, Molecular & Forensic Sciences, Murdoch University, Murdoch, WA 6150, Australia; stevie.ziogos@murdoch.edu.au (S.Z.); ian.dadour@sourcecertain.com (I.R.D.)

^2^ Source Certain, Wangara, DC, WA 6947, Australia

^3^ Physical Evidence, Forensic Science Laboratory, ChemCentre, Bentley, WA 6983, Australia; kpitts@chemcentre.wa.gov.au (K.P.)

^4^ Harry Butler Institute, Murdoch University, Murdoch, WA 6150, Australia

***** Correspondence: p.magni@murdoch.edu.au (P.A.M); Tel.: +61-413955810

Fabric swatches

The morphology of cuts and tears on the field and laboratory fabric swatches was similar and showed a slight unravelling of the yarn ends in the field samples. In general, there was no additional damage detected on any of the fabric swatches by D46 (Figure S1).


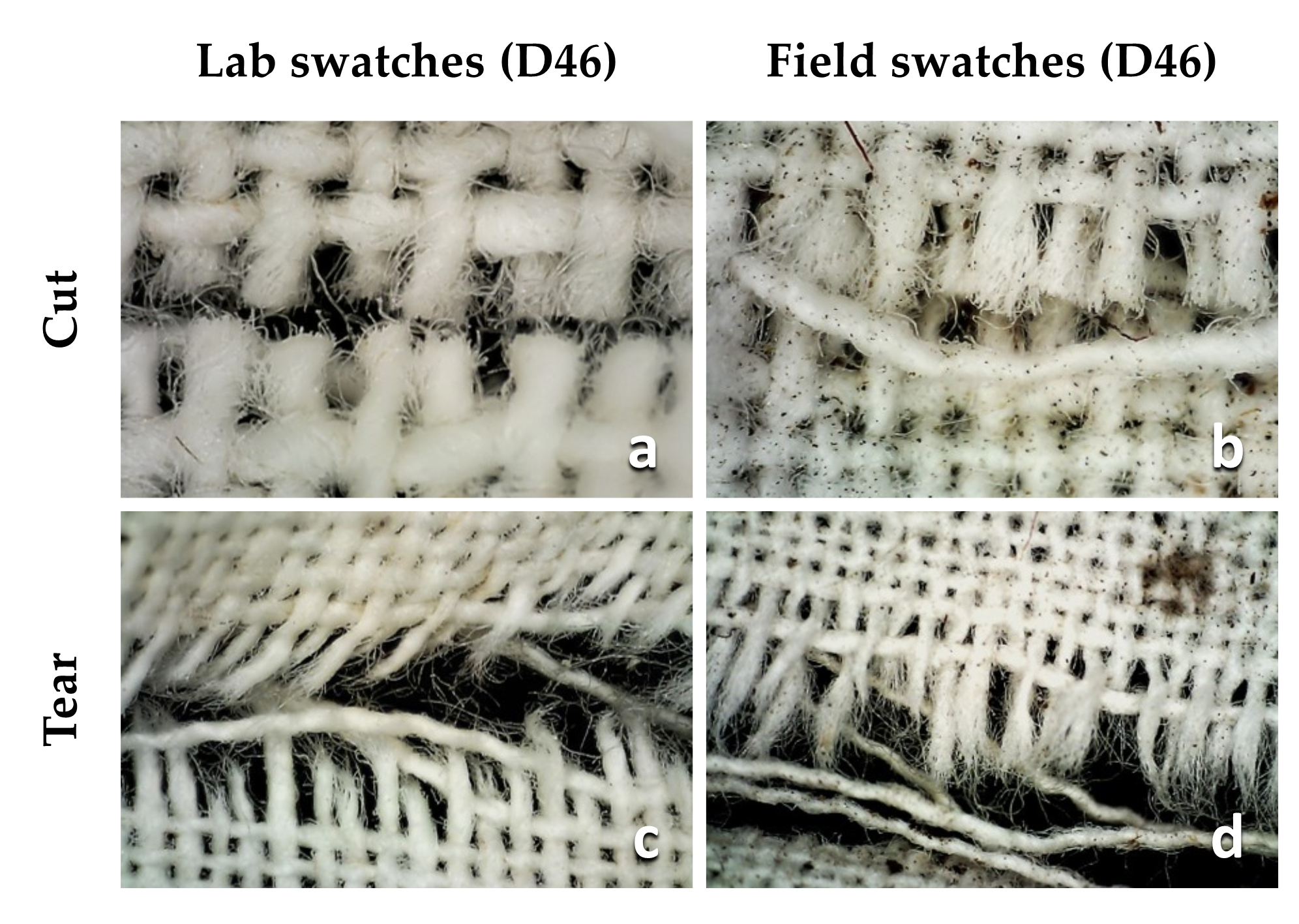


**Figure S1.** Side-by-side comparison of cuts and tears in lab and field swatches on D46. A slight increase in yarn fraying and soiling was observed in the field swatches, but no obvious modification of the damage occurred. **(a)** Cut, cotton lab swatch. **(b)** Cut, cotton field swatch. **(c)** Tear, cotton lab swatch. **(d)** Tear, cotton field swatch.

Comparison of cuts and tears on wrappings to swatches

During the 47-day experiment, modifications were observed in cuts and tears of the piglet wrappings. Among them, cuts that were subsequently stained with biological material underwent the most noticeable alterations, particularly in cotton fabrics. The overall modification of the cuts and tears was attributed to a combination of various factors and events associated with the process of decomposition. These factors encompassed the mechanical stretching of the fabric during the bloating phase, staining from blood and decomposition fluids, the presence of insects attracted to and feeding on the bloodstains resulting from the underlying wounds, mechanical and chemical degradation caused by microbial and fungal growth, as well as the duration of exposure (Figure S2).


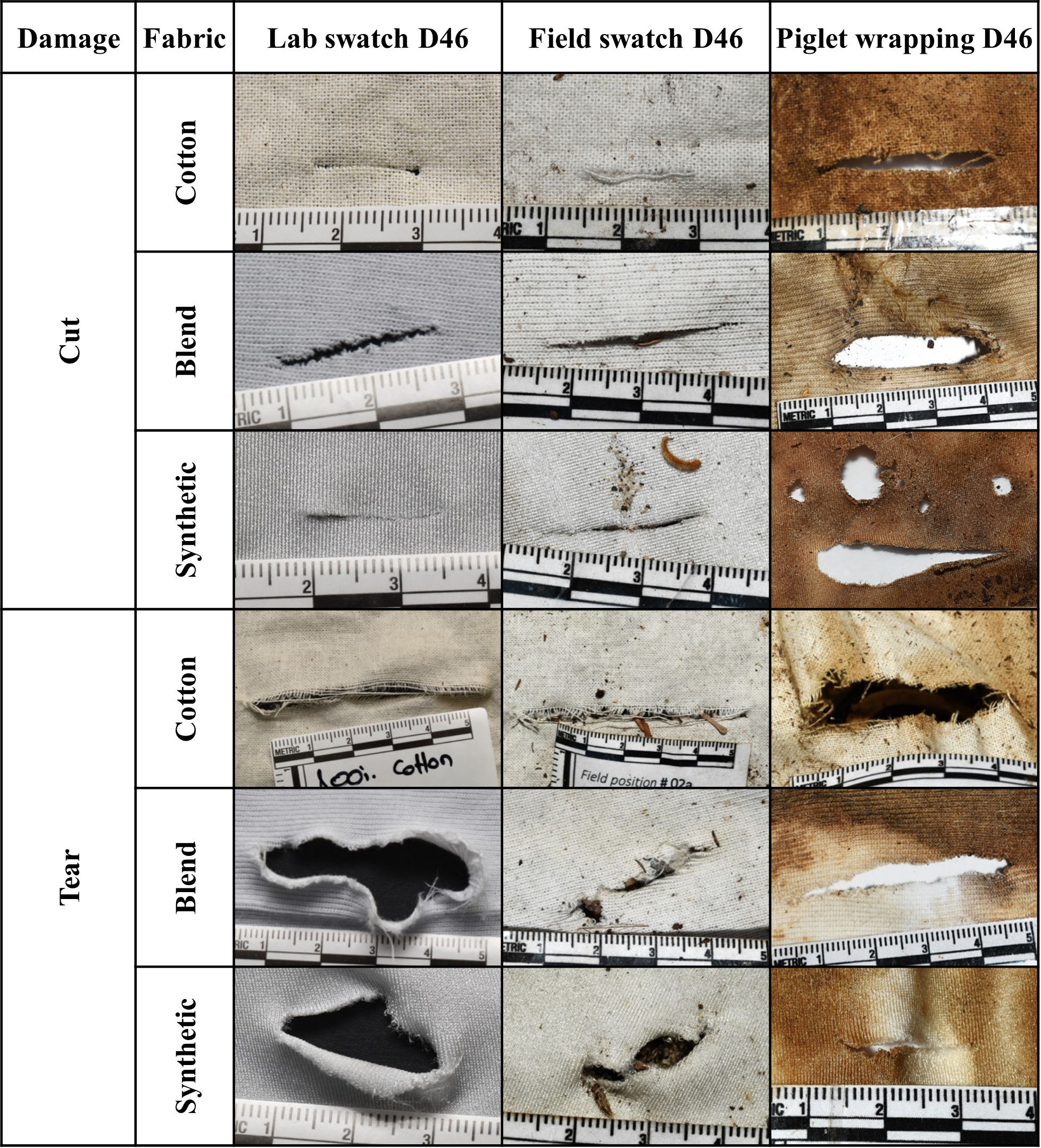


**Figure S2.** Photographs comparing cuts and tears in lab and field swatches and textile damage on wrappings recovered on D46 are shown for the three fabric types.

Piglet decomposition process

Despite the initial size difference of each pig, in both trials the decomposition process attracted a similar group of insects, with unclothed pigs decomposing faster than clothed pigs. Furthermore, unclothed parts of piglets (head) decomposed faster when compared with clothed body parts. The presence of clothing prolonged the wet stage (active and advanced decay) of the body parts protected by the fabric. Clothing also caused an initial reduction in the accessibility of carrion insects to those body parts protected by the fabrics, but such areas soon became of interest to insects because of the higher humidity and temperature, as well as protection from exposure to sunlight and wind. Eventually this facilitated the development of larval masses which aided in the decomposition of the carcass [[1-6](#_ENREF_1)].

Differences in the progress of decomposition were also observed for piglets when wrapped in the different types of fabric. The breathability, and therefore the ability to retain humidity and heat for each of the fabric types, is influenced by the yarn count and weight per square meter (GSM) of the fabric [[7](#_ENREF_7)]. As a consequence, these characteristics have a direct influence on the airspace present in the fabric [[8](#_ENREF_8)]. Cotton has higher breathability compared with other fabric types [[8](#_ENREF_8)] and, in addition, in this experiment cotton had lower GSM when compared with the blended and synthetic fabrics. This resulted in carcasses clothed in cotton having shorter periods of wet decay as compared to piglets clothed in blended and synthetic fabrics.

Bloating

After D2, the shape of the cuts, which was initially linear, acquired a more warped appearance on all fabrics (Figures S3, S4). In addition, the edge and spine characteristics deposited by the knife were affected in most samples. The length of most cuts and tears appeared to have increased by D6 across all fabric types. Cotton fabric was the most affected by stretching, with the front cuts more modified than the rear cuts on the same piglet where the body was more expanded. In an investigation, alterations of the original cut in the fabric can obscure the determination of class characteristics of the potential blade, such as the number of sharp edges and the differentiation between a sharp or serrated blade [[10](#_ENREF_10)]. Tensile stress applied to the inflicted damage also resulted in additional distortion and fraying of the yarns primarily at the edges of the cuts.

Highlights of such alterations are provided in Figures S3, S4 with a particular focus on the modification of size and morphology of the damage during maximum bloating followed by deflation.


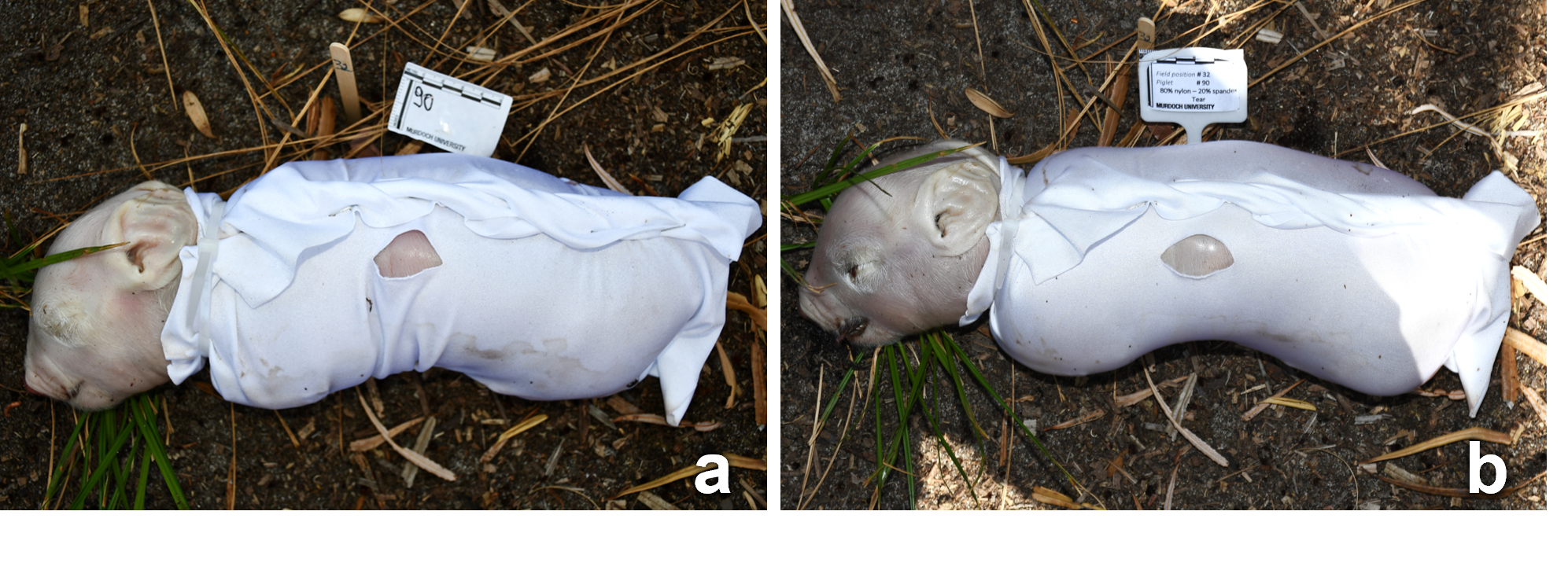


**Figure S3.** Photographs of a wrapped piglet before and during the bloat stage (Tear, Synthetic). **(a)** The torn fabric appears loose before bloating (D2). **(b)** Tensile stress applied to the wrapping due to the expansion of the body during the bloat stage (D5) resulted in fabric stretching. Tensile failure (i.e., further tearing) was observed at the edges of the damage, depending on the stretch capability of the fabric. Consequently, cuts and tears on cotton fabrics primarily exhibited increased end-to-end length compared to blend and synthetic fabric types.


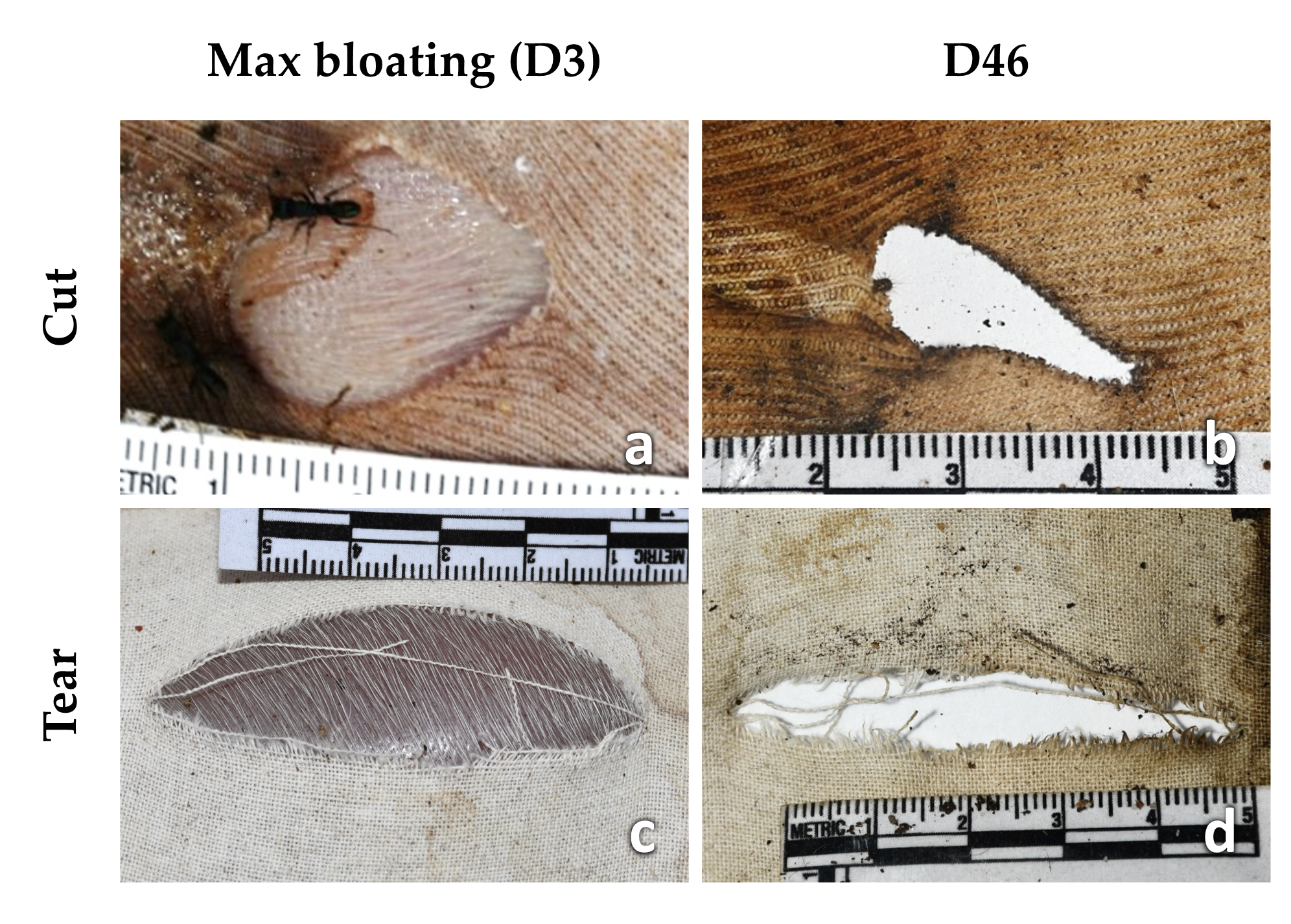


**Figure S4.** Photographs highlighting the modifications to the inflicted fabric damage (cut or tear) on wrappings after the bloat stage and during the process of decomposition. The original size and shape of the damage on the day of maximum bloating and stretch of the damage on D3 are shown in **(a,c)**, and after recovery on D46 in **(b,d)**. After the bloat stage, cuts appeared visibly affected by stretching, resulting in a more warped shape compared to the initial linear shape, as well as the distortion of the blade and spine characteristics from the knife. Tears appeared to be less affected than cuts overall; however, the length of the severances in most samples appeared to have increased.

Staining and weathering

Cut yarns were stained with blood from the underlying wounds, and decomposition material as decomposition progressed. Blood and decomposition material deposits were attractive to insects that were observed to interact with the stained areas of fabric. The bloodstained cut yarns were clumped together, though the flat yarn ends could still indicate cut damage with a sharp blade (Figure S5a). After D11, yarn displacement, fraying, and fractured fibers were observed at the bloodstained edges of cuts and fabric surface of primarily blended and synthetic fabrics. When yarn ends were not completely covered in decomposition material (Figure S5b), it was possible to observe an alteration of the flat yarn ends, a disruption of the alignment of the yarns within the cuts, and the alignment of fibers within the yarns (technically known as “disruption of the planar array”). This was especially evident after D17, with increased fraying, yarn displacement, missing areas of fabric, and additional fiber fractures. Tears represented damage to the fabric, but not to the underlying body, with the fabric remaining unstained until soaked by the body fluids during active decay. Consequently, during the early stages of decomposition, insect colonization was limited in these areas when compared to the wounds underlying cuts. Tears showed alterations due to staining with decomposition material and weathering only after D25, with fraying or clumped yarns and fibers brittle and overly fractured. However, additional fraying due to insect interaction cannot be confirmed, as the observed distortion of the fibers could be due to fraying originally present.

Furthermore, at the end of the wet stages, the drying of decomposition fluids resulted in the loss of fibers, due to their increased brittleness (Figure S5f). Severely modified sections of cuts could potentially imitate characteristics of weathered tears and hinder the determination of characteristics of potential blades, such as the sharpness or blade profile [[11](#_ENREF_11)]. According to a study by Schotman *et al*. [[12](#_ENREF_12)], practitioners were able to evaluate textile damage on fabrics that had been subjected to various weathering factors such as bloodstains and burial. The study showed that these factors did not interfere with the assessment of the textile damage. However, it is worth noting that each factor was tested separately, which does not accurately reflect the decomposition of a body exposed in a natural environment.


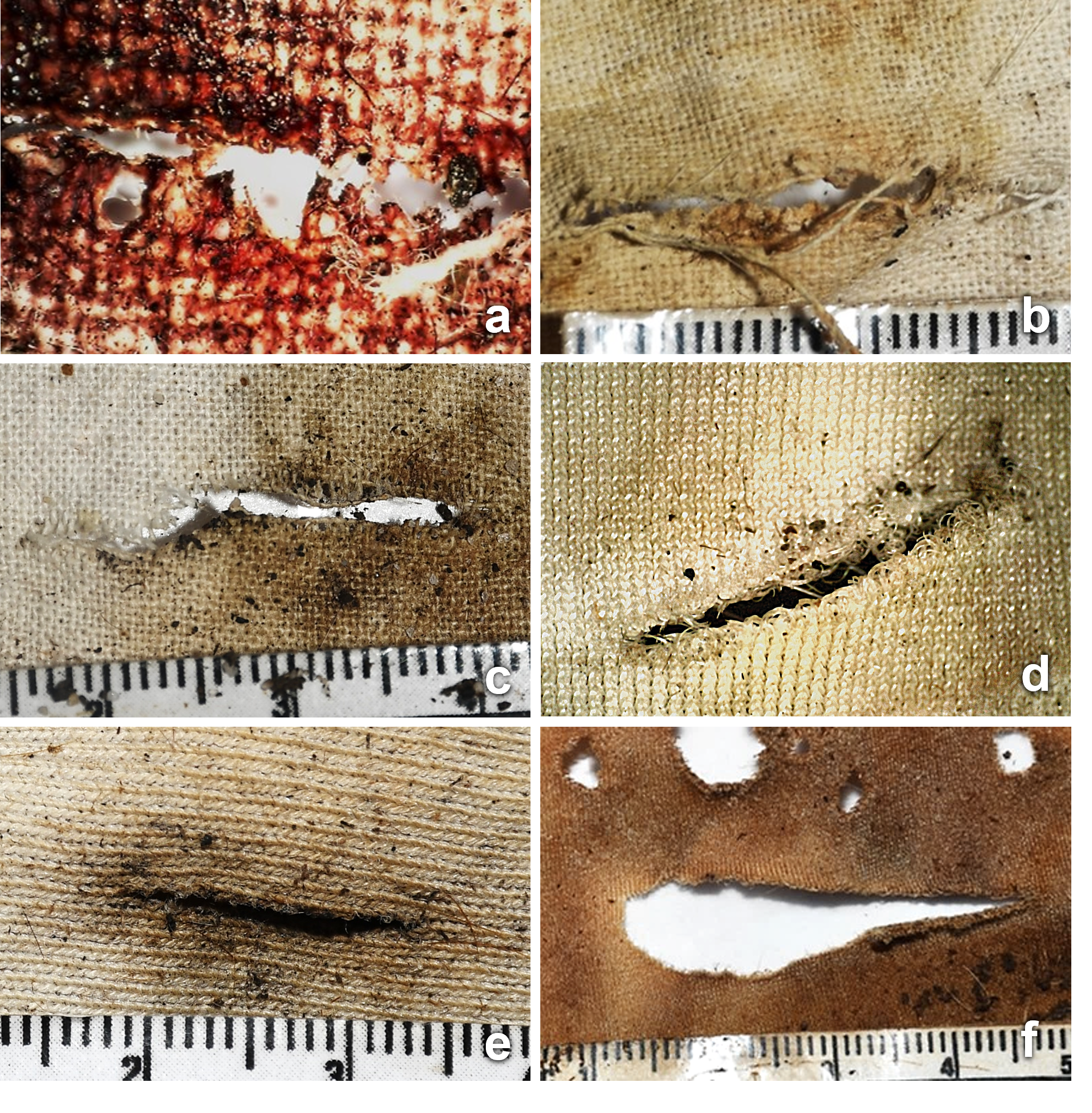


**Figure S5.** Modifications observed on stained cuts recovered on D6, D11, D17, D25, and D46. **(a)** Close-up of bloodstained and clumped cut yarns (Cotton, D6). The even yarn ends indicate a cut made with a sharp blade. **(b)** Cut stained with decomposition material at the edges, partially obscuring the characteristics of the yarn ends, which appear clumped, matted, and deteriorated when not completely obscured (Cotton, D11). **(c)** Stained cut showing tear characteristics on the left end, resulting from tensile stress during bloating (Cotton, D17). **(d)** Cut with increased fraying at the edges and an overall weathered appearance (Synthetic, D25). **(e)** Cut with a darkened appearance where it was previously stained by blood. Additional fraying at the edges and distortion of the stained fabric surface are observed (Blend, D46). **(f)** Modified cut with missing sections, degraded appearance, and additional artifacts near the cut (Synthetic, D46).

Biodegradation of fabrics

Previous research by Ueland *et al.* [[16](#_ENREF_16)] focused on the decomposition of pigs on soil and its effect on cotton clothing associated with the pigs or placed on the soil as controls. On Day 149 of their experiment, the first observations of chemical degradation were on the control T-shirt, some 35 days earlier than the T-shirt worn by pigs. Degradation on the control T-shirt was observed only on areas in contact with the soil, whereas holes on the T-shirt worn by pigs were observed on areas of the fabric not visibly stained with body fluids and not in direct contact with the soil. However, the experiment of Ueland *et al.* [[16](#_ENREF_16)] did not allow for observations on stained areas of fabric along the periphery of each carcass prior to its final removal. In addition, the role of insects was overlooked in this study even though pigs were accessible to carrion insects. The present study demonstrated that fabric degradation was less evident at the interface between the carcass and soil. The fabric in these areas was often embedded with dried decomposition material and soil debris. This observation is in agreement with the findings of Ueland *et al.* [[16](#_ENREF_16)] and Lowe *et al.* [[17](#_ENREF_17)] who recorded that clothing may be preserved when partially or completely buried in soil and in contact with a decomposing pig where decomposition fluids stagnate. Furthermore, holes were observed in the fabric at the interface between the carcass and the soil (Figure S6b) [[18](#_ENREF_18)]. Fly larvae and beetles were frequently observed near these areas, but due to the presence of debris material embedded in the edges of the holes, there is insufficient evidence that these holes were originally caused by insects or that insects use these holes to access the remains. In general, natural fibers like cotton are more susceptible to chemical degradation (Figure S6) due to their high cellulose content and their vulnerability to hydrolysis by bacterial digestion, while polyester and nylon fibers are considerably more resilient [[19](#_ENREF_19),[20](#_ENREF_20)]. These observations underline the complexity of the process of chemical degradation of fabrics, which may be affected by the type and position of possible pre-existing fabric damage, as well as the position of the body during decomposition affecting the distribution of body fluids.


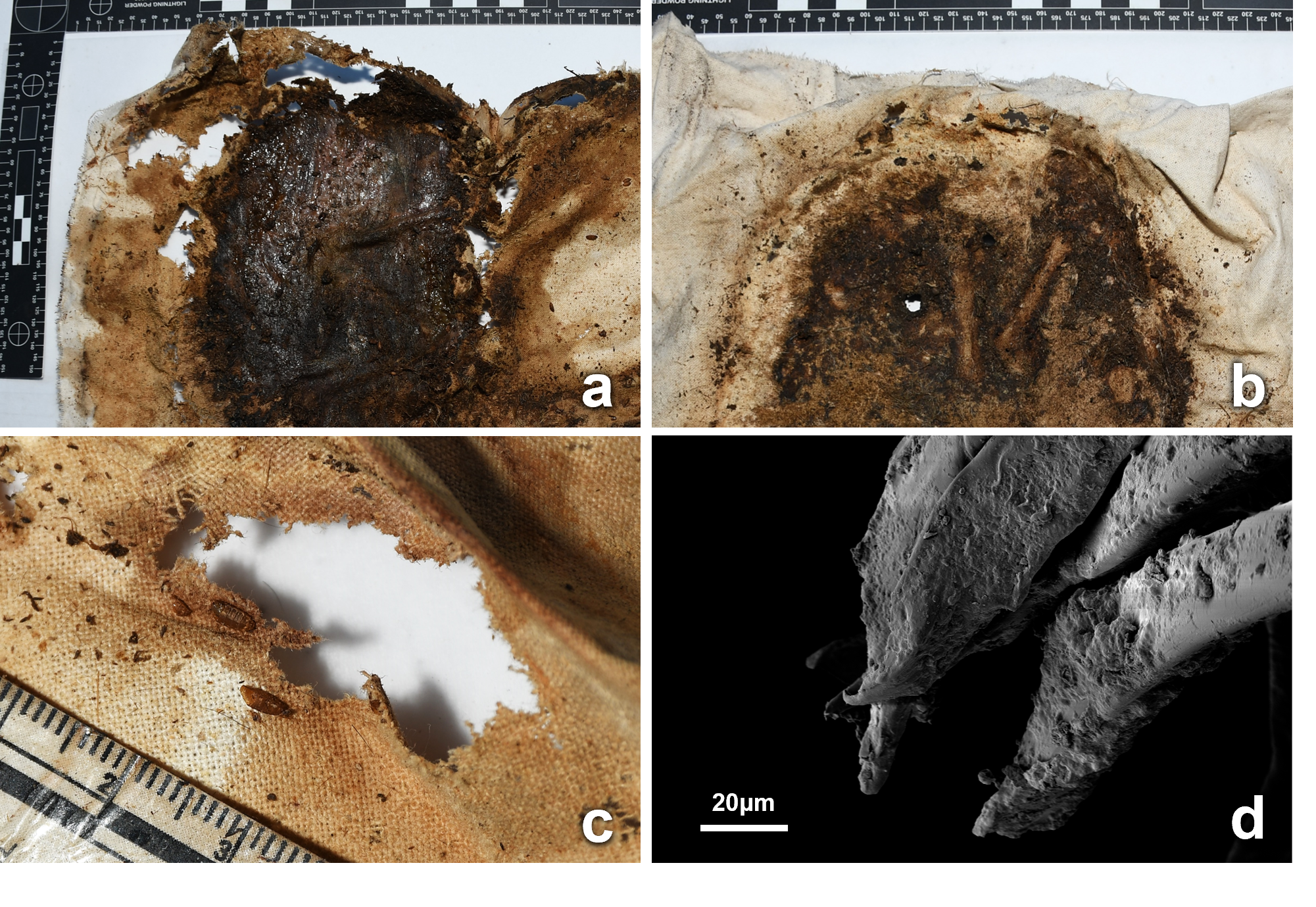


**Figure S6.** Degradation of cotton wrappings. **(a)** Areas of physical and chemical degradation are primarily observed at the periphery of the decomposed carcass, in contact with the soil but not in direct contact with the remains at the time of recovery. The fabric area that was in direct contact with the remains (dark brown) appears relatively preserved (Cotton, D46). **(b)** Large holes with a maximum end-to-end distance of 81 mm, were observed on fabric areas situated directly under the carcass. These holes were embedded with soil, hair, and decomposition material and could have served as access points to the carcass by insects (Cotton, D46). **(c)** Degradation of cotton fabric not in contact with the soil. Insects were commonly observed interacting with the stained areas. Insect remains such as empty pupal cases, indicating prior entomological activity, were often collected from the recovered fabrics (Cotton, D46). **(d)** SEM micrograph of stained nylon fiber specimens taken from synthetic wrapping on D46, showing macroscopically visible signs of degradation. Chemical breakdown of fiber ends with irregular and granular appearance can be observed.


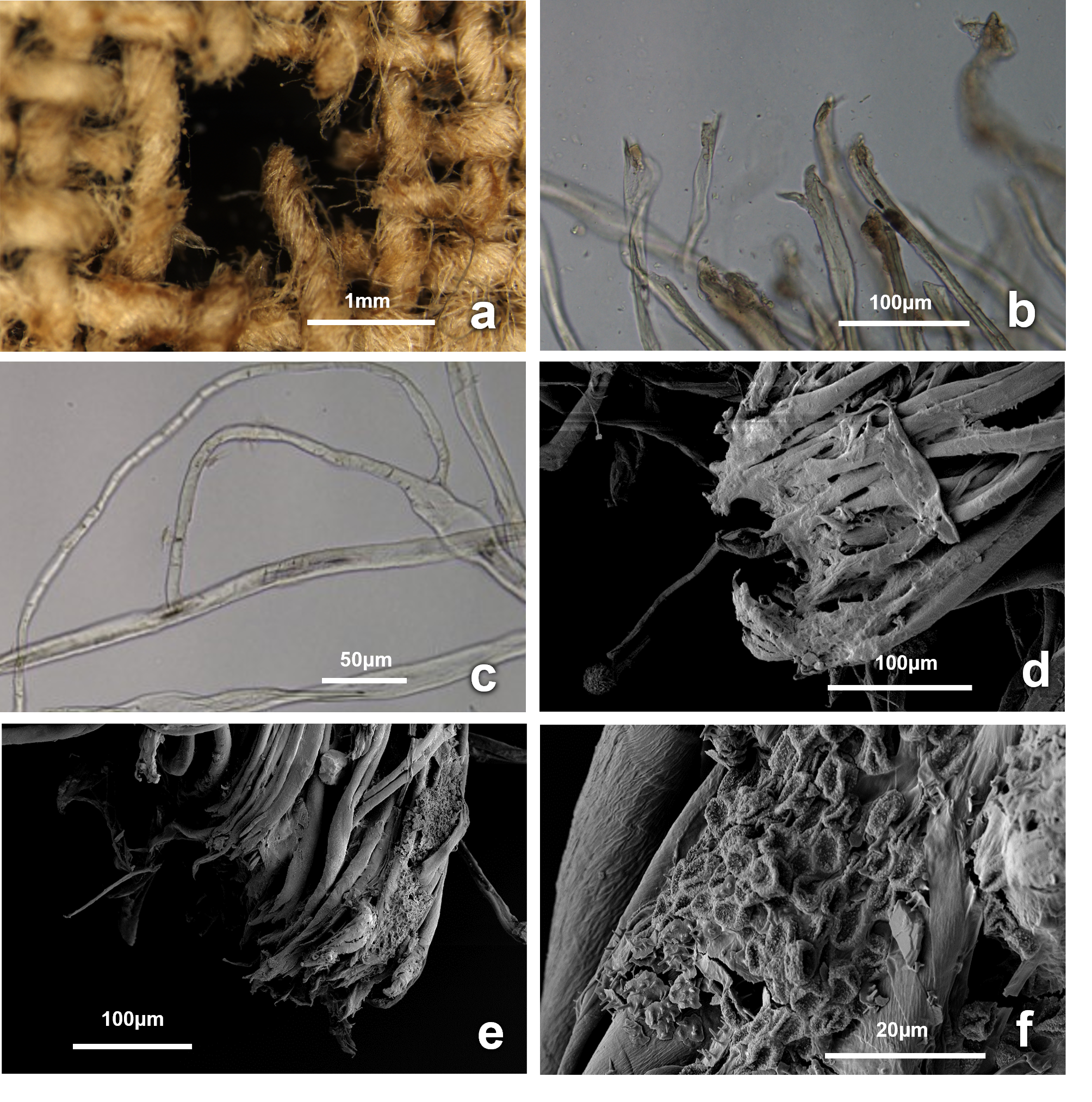


**Figure S7.** Photographs, micrographs and electron micrographs of artifact-damaged yarns and fibers. **(a)** Close-up of a newly observed artifact on cotton fabric recovered on D6, showing an overall darkened appearance formed by missing sections of yarns. Macroscopically, the yarns had minimal fraying, and appeared clumped, brittle, and with missing sections. Fiber specimens taken from this artifact showed darkened fiber ends with cracks and splitting **(b)** at the ends and **(c)** along the fiber axes. **(d,e)** SEM micrographs showing collective chemical breakdown mostly at the fiber ends but also along the axes. **(f)** Fruiting bodies, fungal cells, spores, and hyphae were apparent on yarns and fibers, and the appearance of the yarn was consistent with the effect of biodeterioration.

Ants and opportunistic insects


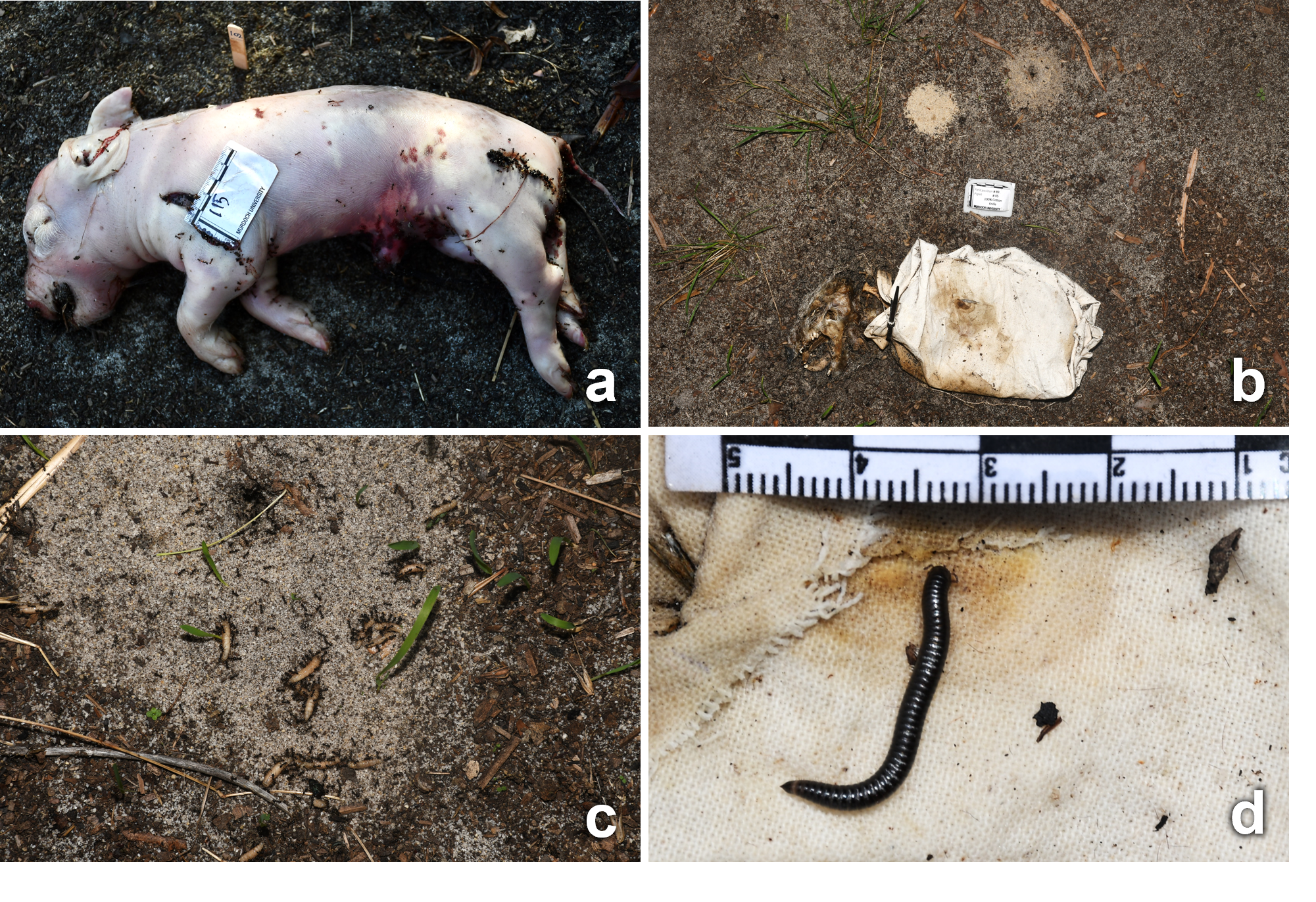


**Figure S8.** Ants and opportunistic insects observed from D2 and throughout the trial. **(a)** Ants were observed on D2 at the bleeding wounds, ears, and genitals of piglets (when accessible). **(b)** New ant nests were found close to several carcasses (D17). **(c)** Ants were frequently observed removing larvae from the carcasses (D17). **(d)** Millipedes and other insects were also observed feeding on biological fluids that had seeped into the fabrics.

Effect of insect activity on fabrics


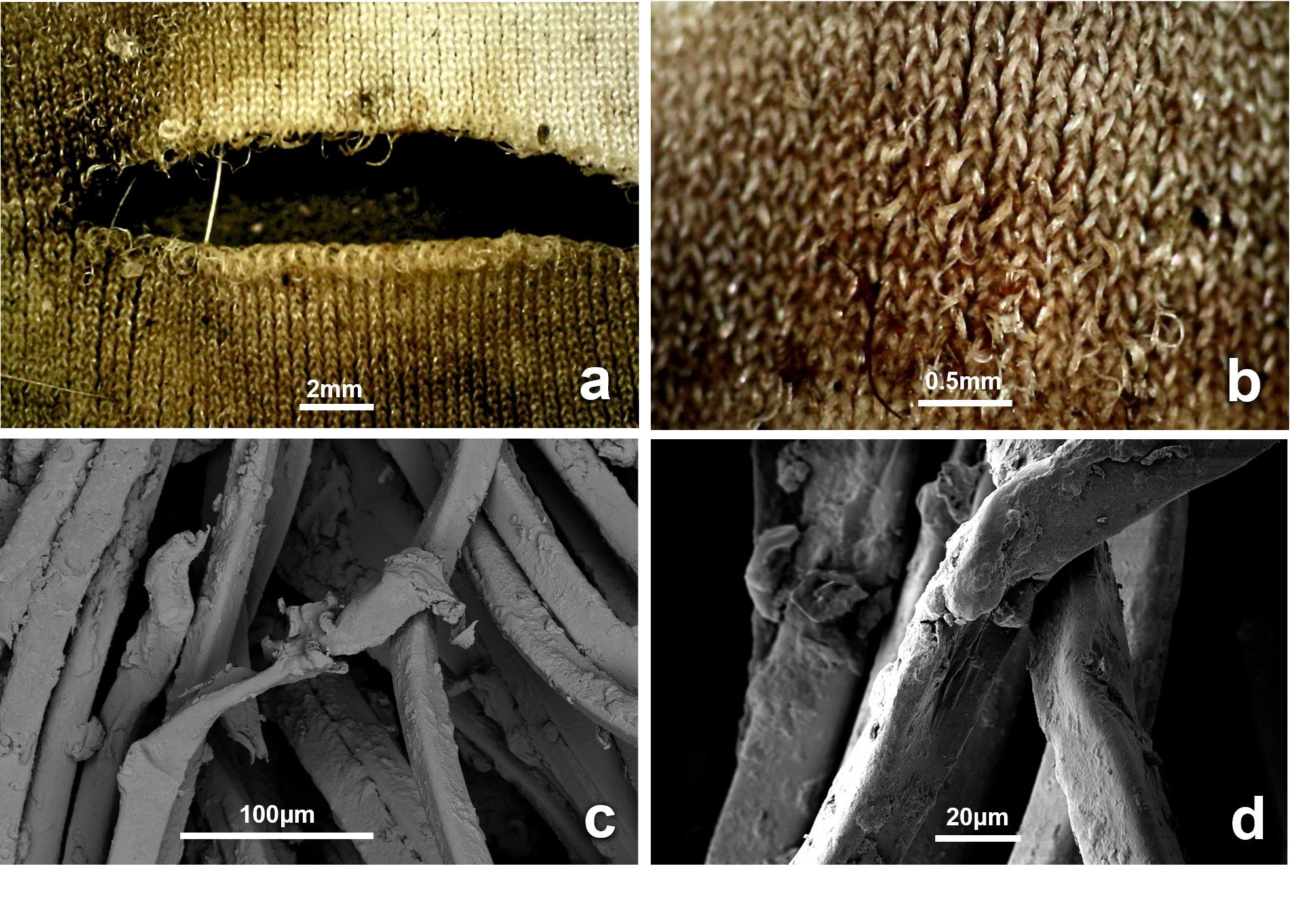


**Figure S9.** Distortion of stained fabric surface (Cut, Synthetic, D25). **(a)** Yarns and fibers pulled out of structure in the stained area. Closer microscopic examination **(b)** and SEM analysis **(c, d)** of the affected fabric areas revealed distortion at the fiber structural level and fractures that could have resulted from insect feeding.


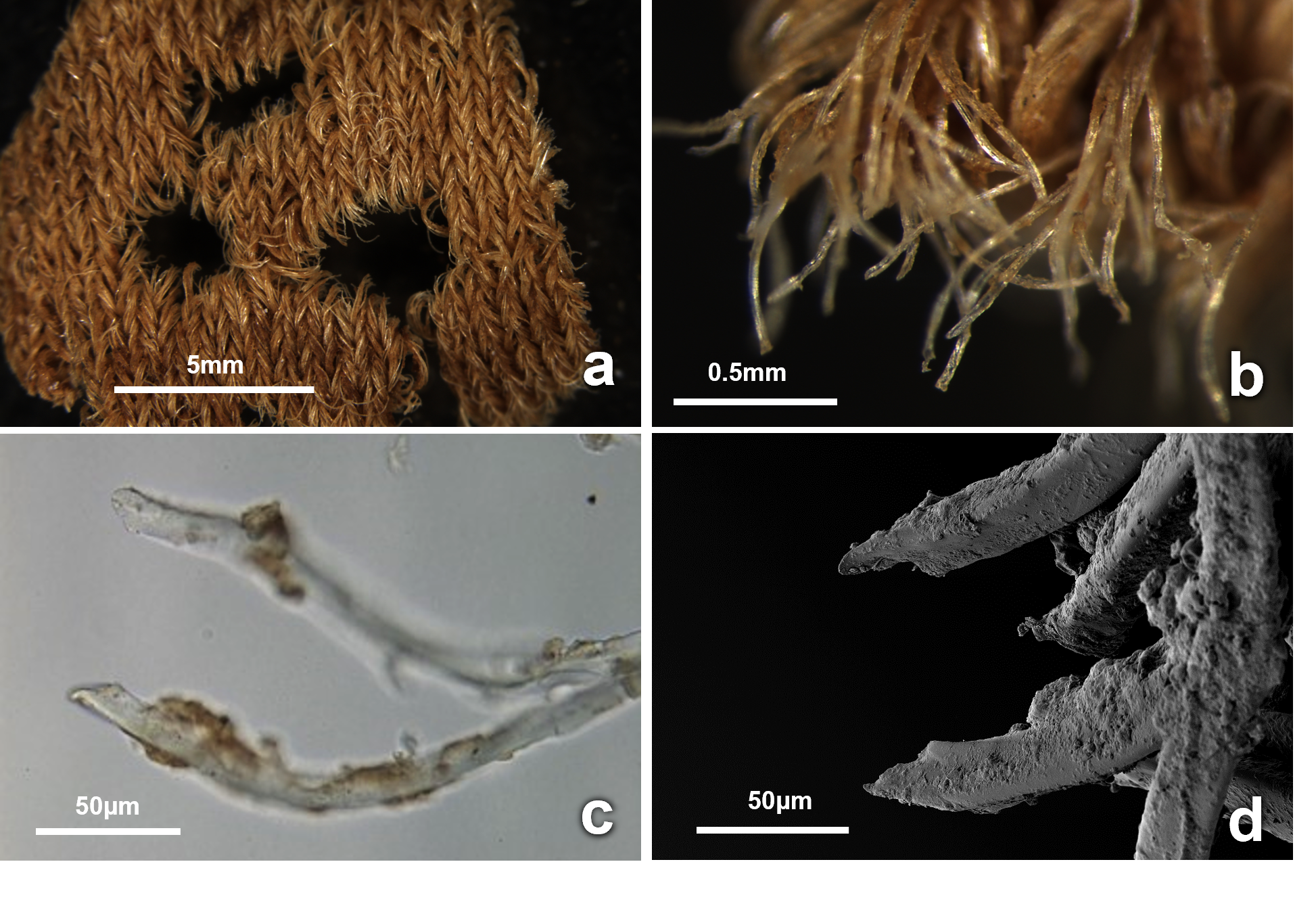


**Figure S10.** New artifacts (holes) with distortion of the fabric surface on a stained and previously intact area (Synthetic, D46) **(a)**. The fiber ends showed brittle fractures **(b)**, often concave-shaped, at both the fiber ends and along the fiber axes **(c, d)**.

References

1. Hewadikaram, K.A.; Goff, M.L. Effect of carcass size on rate of decomposition and arthropod succession patterns. *Am J Forensic Med Pathol* **1991**, *12*, 235-240, doi:10.1097/00000433-199109000-00013.

2. Simmons, T.; Cunliffe, R.; Moffatt, C. Debugging Decomposition Data-Comparative Taphonomic Studies and the Influence of Insects and Carcass Size on Decomposition Rate. *Journal of forensic sciences* **2009**, *55*, 8-13, doi:10.1111/j.1556-4029.2009.01206.x.

3. Voss, S.C.; Cook, D.F.; Dadour, I.R. Decomposition and insect succession of clothed and unclothed carcasses in Western Australia. *Forensic Science International* **2011**, *211*, 67-75.

4. Kelly, J.A.; Van Der Linde, T.C.; Anderson, G.S. The influence of clothing and wrapping on carcass decomposition and arthropod succession during the warmer seasons in central South Africa. *Journal of forensic sciences* **2009**, *54*, 1105-1112.

5. Goff, M. Problems in Estimation of Postmortem Interval Resulting from Wrapping of the Corpse: A Case Study from Hawaii'. *J Agri Entomol* **1992**, *9*.

6. Card, A.; Cross, P.; Moffatt, C.; Simmons, T. The Effect of Clothing on the Rate of Decomposition and Diptera Colonization on Sus scrofa Carcasses. *Journal of Forensic Sciences* **2015**, *60*, 979-982, doi:<https://doi.org/10.1111/1556-4029.12750>.

7. Bonaldi, R.R. Functional finishes for high-performance apparel. In *High-Performance Apparel*, McLoughlin, J., Sabir, T., Eds. Woodhead Publishing: 2018; <https://doi.org/10.1016/B978-0-08-100904-8.00006-7pp>. 129-156.

8. Hunter, L.; Fan, J. Waterproofing and breathability of fabrics and garments. In *Engineering Apparel Fabrics and Garments*, Fan, J., Hunter, L., Eds. Woodhead Publishing: 2009; <https://doi.org/10.1533/9781845696443.283pp>. 283-308.

9. Komar, D.; Beattie, O. Postmortem insect activity may mimic perimortem sexual assault clothing patterns. *Journal of forensic sciences* **1998**, *43*, 792.

10. Kemp, S.E. Forensic Analysis of Sharp Weapon Damage to Textile Products. In *Forensic Textile Science*, Carr, D., Ed. Woodhead Publishing: 2017; <https://doi.org/10.1016/B978-0-08-101872-9.00005-4pp>. 71-97.

11. Nichols-Drew, L.; Armitage, R.; Hillman, R.; Sheridan, K.J.; Farrugia, K.J. On a knife edge: A preliminary investigation of clothing damage using rounded-tip knives. *Science & Justice* **2020**, *60*, 495-503, doi:<https://doi.org/10.1016/j.scijus.2020.08.002>.

12. Schotman, T.; Soedhoe, R.; Van der Weerd, J. Toward a systematic classification of textile damages. *Forensic Sci Rev* **2018**, *30*, 51-75.

13. Monahan, D.L.; Harding, H.W.J. Damage to Clothing—Cuts and Tears. *Journal of Forensic Sciences* **1990**, *35*, 12903J, doi:10.1520/jfs12903j.

14. Kemp, S.E.; Carr, D.J.; Kieser, J.; Niven, B.E.; Taylor, M.C. Forensic evidence in apparel fabrics due to stab events. *Forensic Sci Int* **2009**, *191*, 86-96, doi:10.1016/j.forsciint.2009.06.013.

15. Robertson, J.; Roux, C.; G., W.K. *Forensic Examination of Fibers*, Third ed.; Boca Raton: Taylor and Francis: Boca Raton, FL, 2017.

16. Ueland, M.; Nizio, K.D.; Forbes, S.L.; Stuart, B.H. The interactive effect of the degradation of cotton clothing and decomposition fluid production associated with decaying remains. *Forensic Science International* **2015**, *255*, 56-63.

17. Lowe, A.C.; Beresford, D.V.; Carter, D.O.; Gaspari, F.; O’Brien, R.C.; Stuart, B.H.; Forbes, S.L. The effect of soil texture on the degradation of textiles associated with buried bodies. *Forensic Science International* **2013**, *231*, 331-339, doi:<https://doi.org/10.1016/j.forsciint.2013.05.037>.

18. Byrd, J.H.; Tomberlin, J.K. *Forensic entomology: the utility of arthropods in legal investigations*, Third ed.; CRC Press, Taylor & Francis Group: Boca Raton, FL, 2020.

19. Janaway, R.C. Degradation of clothing and other dress materials associated with buried bodies of archaeological and forensic interest. In *Advances in Forensic Taphonomy*, CRC Press: 2001; pp. 379-402.

20. Mills, J.; Klausmeier, R.E. The biodeterioration of synthetic polymers and plasticizers. *C R C Critical Reviews in Environmental Control* **1974**, *4*, 341-351, doi:10.1080/10643387409381618.
